# High Salt Inhibits Tumor Growth by Enhancing Anti-tumor Immunity

**DOI:** 10.3389/fimmu.2019.01141

**Published:** 2019-06-04

**Authors:** Ralf Willebrand, Ibrahim Hamad, Lauren Van Zeebroeck, Máté Kiss, Kirsten Bruderek, Anneleen Geuzens, Dries Swinnen, Beatriz Fernandes Côrte-Real, Lajos Markó, Els Lebegge, Damya Laoui, Josephine Kemna, Thomas Kammertoens, Sven Brandau, Jo A. Van Ginderachter, Markus Kleinewietfeld

**Affiliations:** ^1^VIB Laboratory of Translational Immunomodulation, VIB Center for Inflammation Research, University of Hasselt, Campus Diepenbeek, Hasselt, Belgium; ^2^Cellular and Molecular Immunology Lab, Vrije Universiteit Brussel, Brussels, Belgium; ^3^Myeloid Cell Immunology Lab, VIB Center for Inflammation Research, Brussels, Belgium; ^4^Research Division, Department of Otorhinolaryngology, West German Cancer Center, University Hospital Essen, Essen, Germany; ^5^Experimental and Clinical Research Center, A Joint Cooperation of Max Delbrück Center for Molecular Medicine and Charité University Medicine Berlin, Berlin, Germany; ^6^Institute of Immunology, Charité - Universitätsmedizin Berlin, Berlin, Germany; ^7^Max Delbrück Center for Molecular Medicine, Berlin, Germany

**Keywords:** cancer, dietary factor, MDSC, cancer immunotherapy, sodium chloride (dietary)

## Abstract

Excess salt intake could affect the immune system by shifting the immune cell balance toward a pro-inflammatory state. Since this shift of the immune balance is thought to be beneficial in anti-cancer immunity, we tested the impact of high salt diets on tumor growth in mice. Here we show that high salt significantly inhibited tumor growth in two independent murine tumor transplantation models. Although high salt fed tumor-bearing mice showed alterations in T cell populations, the effect seemed to be largely independent of adaptive immune cells. In contrast, depletion of myeloid-derived suppressor cells (MDSCs) significantly reverted the inhibitory effect on tumor growth. In line with this, high salt conditions almost completely blocked murine MDSC function *in vitro*. Importantly, similar effects were observed in human MDSCs isolated from cancer patients. Thus, high salt conditions seem to inhibit tumor growth by enabling more pronounced anti-tumor immunity through the functional modulation of MDSCs. Our findings might have critical relevance for cancer immunotherapy.

## Introduction

The balance between pro- and anti-inflammatory cells and signals is critical for preserving immune homeostasis and a disturbed immune cell balance is believed to contribute to autoimmunity and cancer. Recent data have demonstrated that a high salt diet (HSD) could influence the immune cell balance toward a pro-inflammatory state, where the induction of pro-inflammatory cells, such as T helper 17 cells (T_H_17) and M1-like macrophages is promoted and the function of anti-inflammatory cells, such as M2-like macrophages and regulatory T cells (T_regs_) is impaired (1–4). High salt intake is a ubiquitous phenomenon of Western diets and is indeed implicated in a plethora of diseases like cardiovascular and metabolic as well as autoimmune diseases (5, 6). Particularly the pro-inflammatory effects of a HSD are believed to be associated with autoimmune diseases like e.g., multiple sclerosis (MS) and inflammatory bowel diseases (IBD) (7). These pro-inflammatory effects of high salt on the immune cell balance raise the question if high salt conditions could also affect anti-tumor immunity and cancer.

The immune system is able to recognize neoplasms and after therapeutic intervention it can also attack and eradicate tumors as recent advances in the field of cancer immunotherapy (e.g., immune checkpoint inhibition) have shown. Indeed, immunotherapy is one of the most promising approaches to treat cancer (8, 9). However, a major obstacle for successful cancer immunotherapy is the highly immuno-suppressive environment induced by many tumors. It is well-documented that the tumor microenvironment frequently induces an immune protective and tolerogenic environment by e.g., promoting the induction of various immune suppressive cell types or by inducing the expression of immune suppressive cytokines (10–14).

Important players in the tumor microenvironment are myeloid cells, which can support tumor growth by providing growth factors, such as vascular endothelial growth factor (VEGF) (15) and additionally can be strongly immunosuppressive. One of these tumor promoting myeloid cell types are myeloid-derived suppressor cells (MDSCs) (16–18). MDSCs are a classically defined as a heterogeneous population of immature myeloid cells that fail to terminally differentiate and exert a strong immune suppressive potential in mice and humans. The induction of MDSCs from myeloid progenitors occurs in the bone marrow (BM) and spleen usually in the context of chronic inflammation and often leads to an accumulation of these cells in the periphery in cancerous conditions. Here they can inhibit various other immune cells like T and B cells, dendritic cells (DC) and natural killer cells (NK) and thereby contribute to an immune suppressive state using different molecular mechanisms of suppression. However, also in the absence of T cells, GR1 positive myeloid cells can support tumor growth (19) e.g., by the promotion of tumor angiogenesis (15). The presence of MDSCs in cancer patients is associated with a poor disease prognosis and tumor recurrence (17, 20). MDSCs can be further subdivided based on their origin and phenotype into granulocytic or polymorphonuclear MDSCs (PMN-MDSCs) and monocytic (M-MDSCs) (17, 21). Both MDSC subsets can be frequently detected in BM, spleen, blood and tumor tissues of cancer patients. In mice PMN-MDSCs are defined as CD11b^+^Ly6G^high^Ly6C^low^, whereas M-MDSCs are defined as CD11b^+^Ly6G^−^Ly6C^high^ cells, although both marker combinations are not entirely specific and can also include neutrophils or classical monocyte populations (17). In humans PMN-MDSCs are characterized by CD11b^+^CD14^−^CD15^+^HLA-DR^−^ or CD11b^+^CD14^−^CD66b^+^ expression and M-MDSCs are CD11b^+^CD14^+^CD15^−^HLA-DR^low/−^ cells (17). MDSCs further show a high grade of plasticity. They can react to environmental triggers like altered cytokine milieus or hypoxia and adapt their phenotype and function accordingly or can even differentiate into cells with pro-inflammatory potential (18, 22). However, how and if MDSCs react to changes in the ionic microenvironment as e.g., found in tumor tissues through increased necrosis (23) or through induction by HSD (24, 25) is unknown.

Since high salt affects various adaptive and innate immune cells it is plausible that MDSCs may also react to elevated Na^+^ concentrations. Considering the immune stimulatory effects of high salt, these conditions may be favorable for enhanced tumor immunology by boosting pro-inflammatory effector cells and blocking anti-inflammatory cells. We therefore sought to analyze the impact of HSD on tumor growth in murine tumor transplantation models. Here we show that a HSD significantly reduces tumor growth in two independent tumor transplantation models. The effect seems to be largely dependent on myeloid cells by impacting MDSC function and thereby leading to enhanced anti-tumor immunity. Thus, our study identified a novel effect of how high dietary salt intake could modify innate immune reactions by modulating murine and human MDSC function. These data may offer novel strategies for improving cancer immunotherapies.

## Materials and Methods

### Mice

C57BL/6 mice were purchased from Charles River and housed in the facility of the University of Hasselt under standardized conditions. Further, C57BL/6 mice were purchased from Janvier and housed in the facility of Vrije Universiteit Brussel (VUB) under standardized conditions. *RAG2*^**−/−**^ mice were kindly provided by Thomas Blankenstein. Animal studies were approved by the ethics committees of animal studies at the University of Hasselt (201738) and VUB (14-220-26).

### Diet and Tumor Inoculation

Mice were either fed a normal diet (Control group) containing 0.5% NaCl or sodium enriched diet (HSD group) containing 4% NaCl as well as 1% NaCl enriched tap water for 2 weeks before tumor inoculation. In some experiments, diet switch was started directly before tumor inoculation. Both diets were purchased from SSNIFF (Ctrl: E15430-04, HSD: E15431-34; Soest, Germany). Mice were maintained on the respective diet during the course of the experiment. B16F10 melanoma cells (ATCC) were cultured in DMEM (Sigma Aldrich) supplemented with 10% FCS (Gibco) and Penicillin/Streptomycin (Gibco) and Lewis Lung carcinoma (LLC) cells (ATCC) were cultured in RPMI (Lonza) supplemented with 10% FCS and Penicillin/Streptomycin. Cells were maintained mycoplasma free, tested continuously by HEK-blue mycoplasma detection (Invivogen). Tumor cells were subcutaneously injected in the left abdominal flank (LLC at 1 × 10e6 expose 6 and B16F10 at 2 × 10e5 expose 5 cells/mouse). Tumor growth was monitored three times per week by using a caliper. Tumor volume was calculated with the ellipsoid formula (π/6^*^a^*^b^*^c) as described before (26).

### Flow Cytometry and Preparation of Single Cell Suspensions

Blood was taken by tail vein puncture. Spleens and lymph node(s) were mashed through a 70 μm cell strainer. Tumor tissue was minced and subjected to digestion cocktail containing collagenase at 500 μg/ml and DNAse at 40 units/ml for 30 min at 37°C and passed through a 70 μm cell strainer. Single cell suspensions were subjected to red blood cell lysis (eBioscience). Cells were washed with MACS buffer (0.5% BSA 2 mM EDTA) and subjected to flow cytometry (further named FACS) staining protocol or resuspended in complete RPMI medium and restimulated with 50 ng/ml phorbol 12-myristate 13-acetate (PMA) and 250 ng/ml Ionomycin (Sigma) in the presence of GolgiPlug (BD) for 5 h to detect cytokines by FACS. Single cell suspensions were firstly stained with fixable Live/Dead cell kit (Thermo Fisher Scientific) for 10 min at room temperature. Cells were then incubated with antibody cocktails for 30 min at 4°C in MACS buffer. Intracellular staining was performed using the FoxP3 staining kit (eBioscience) according to the manufacturers protocol. The following antibodies were used: CD3 (Biolegend or eBioscience, 17A2), CD4 (Biolegend or BD Pharmingen, RM4-5), CD8 (eBioscience, 53-6.7), CD11b (eBioscience, M1/70), CD25 (BD Pharmingen, PC61), CD44 (eBioscience, IM7), CD45.2 (BD, 104), CD62L (eBioscience, MEL-14), CD183 (Biolegend, CXCR3-173), CD192 (Biolegend, SA203G11), CD196 (Biolegend, 29-2LI7), CD274 (Biolegend, 10F.9G2), CD279 (BD, J43), FoxP3 (eBioscience, FJK-16s), F4/80 (eBioscience, BM8), I-A/I-E (BD, M5/114.15.2), IFNγ (eBioscience, XMG1.2), IL-9 (Biolegend, RM9A4), IL-10 (eBioscience, JES5-16E3), IL-17 (eBioscience, eBio17B7), Ly6C (Biolegend, HK1.4), Ly6G (Biolegend, 1A8), Siglec-F (BD, E50-2440) and TNFα (Biolegend, MP6-XT22), Eomes (eBioscience, Dan11mag), T-bet (Biolegend, 4B10), CD19 (Biolegend, MB19-1), CD5 (Biolegend, 53-7.3). MDSCs were defined as CD11b^+^Ly6C^high^Ly6G^−^ (M-MDSC) and as CD11b^+^Ly6C^med^Ly6G^high^ (PMN-MDSC) pre gated on CD45.2^+^ live cells excluding doublets and dead cells. Cells were acquired on a BD FACS-Fortessa instrument (BD) and analyzed using FlowJo V.10.1 software (FlowJo LLC) and by using FlowSOM.

### FlowSOM Analysis

FACS data was manually gated on single live and/or CD11b^+^ or CD4^+^ T cells and later exported as FCS files in FlowJo V.10.1 (FlowJo, LLC). The automated analysis of exported FCS files was done by using FlowSOM algorithm, a R bioconductor package that uses self-organizing maps for dimensional reduction visualization of flow cytometry data (27). All data was concatenated scaled and logical transformed on import. Cells were assigned to a Self-Organizing Map (SOM) with a 10 × 10 grid, grouping similar cells into 100 nodes. Each node in the FlowSOM tree gets a score indicating its correspondence with this requested cell profile. To visualize similar nodes in branches, a minimal spanning tree (MST) was constructed and cell counts were log scaled and nodes with similar expression markers were clustered within metaclusters. The FlowSOM algorithm was run three times to ensure reproducibility of the results. Comparisons between groups (HSD and Ctrl) were performed using a Mann-Whitney test by computing the mean percentage per sample group in each cluster and by testing statistical significance on every node within metaclusters. *P*-values were two-sided and analysis was performed using RStudio (version 3.4.4).

### Apoptosis Assay

B16F10 and LLC cells were cultured under 40 mM NaCl concentration or 80 mM Mannitol (Sigma Aldrich) as an osmolyte control and additional control cells were cultured in medium only. Cells were harvested after 48 h by trypsinization and resuspended in AnnexinV binding buffer (0.01 M Hepes, 0.14 M NaCl, 2.5 mM CaCl_2_). AnnexinV-FITC (BD) was added for 30 min. Propidium iodide (PI) was added at 1 μg/ml shortly before acquisition on a FACS Calibur instrument (BD).

### ELISA

Serum samples from tumor-bearing control and HSD fed mice were subjected to TNFα-, IL17A, IL-10-, and IFNγ- specific ELISA. All ELISA-kits were purchased from RD-Systems and performed according to the manufacturers protocol. Finally, wells were incubated with the horseradish peroxidase substrate o-phenylenediamine dihydrochloride (OPD) (Thermo Scientific) and optical density measurement was done on the iMark microplate reader (Biorad) with a 450 nm wavelength filter.

### Antibody Depletion

Anti-Gr-1 (clone RB6-8C5) depletion antibody was purchased from BioXCell (West Lebanon, NH). 200 μg were i.p. injected 4 days after tumor injection and later on every second day. Control mice received PBS injection at the same time. Depletion efficiency was monitored by FACS analysis of blood samples using antibodies against CD11b and Ly6-C.

### Quantitative Real-Time PCR

Tumor or spleen tissue was placed in RLT buffer containing b-mercaptoethanol and shredded in a Tissue-lyser (Qiagen). RNA was isolated from the lysates using the RNeasy Kit (Qiagen). RNA was reversely transcribed using the Quanta cDNA Kit (Quanta Biosciences). Quantitative real-time PCR was performed with the Power up SYBR Green Master Mix (Applied Bioscience). Samples were measured on the Step ONE Plus RT-PCR machine (Applied Biosciences). The following primers were used: *Tnf*α forward: 5′-GAGCAATGACTCCAAAGTAG-3′, *Tnf*α reverse: 5′-CGTAGCAAACCACCAAGTGG-3′, *Ifn*γ forward: 5′-AAAGAGATAATCTGGCTCTGC-3′, *Ifn*γ reverse: 5′-GCTCTGAGACAATGAACGCT-3′, *Nos2* forward: 5′-CCCTTCAATGGTTGGTACATGG-3′, *Nos2* reverse: 5′-ACATTGATCTCCGTGACAGCC-3′, *IL-10* forward: 5′-ATAACTGCACCCACTTCCCA-3′, *IL-10* reverse: 5′-GGGCATCACTTCTACCAGGT-3′, *Csf2* forward: 5′-TTTACTTTTCCTGGGCATTG-3′, *Csf2* reverse: 5′-TAGCTGGCTGTCATGTTCAA-3′, *Sgk1* forward: 5′-CCAAACCCTCCGACTTTCAC-3′, *Sgk1* reverse: 5′-CCTTGTGCCTAGCCAGAAGAA-3′, *Il17a* forward: 5′-ATCCCTCAAAGCTCAGCGTGTC-3′, *Il17a* reverse: 5′-GGGTCTTCATTGCGGTGGAGAG-3′, *Pbgd* forward: 5′ TGGTTGTTCACTCCCTGAAGG-3′ and *Pbgd* reverse: 5′-AAAGACAACAGCATCACAAGGGT-3′, *Hprt* forward: 5′-GTTGGATACAGGCCAGACTTTGTT-3′ and *Hprt* reverse: 5′-GAGGGTAGGCTGGCCTATAGGCT-3′. Data was analyzed by 2^−ΔΔ*Ct*^ method.

### Murine MDSC Isolation and Suppression Assay

Subcutaneous LLC tumors were excised and treated with 10 U/ml collagenase I, 400 U/ml collagenase IV and 30 U/m1 DNase I (Worthington) for 30 min at 37°C. Tumors and spleens were squashed and filtered. Red blood cells in spleen and tumor cell suspensions were removed using erythrocyte lysis buffer. To purify MDSCs, CD11b^+^ cells were enriched by using anti-CD11b microbeads (Miltenyi Biotec). MDSCs were sorted from CD11b^+^ cells using FACS Aria II (BD Biosciences) (Supplementary Figure 10). Post sort analysis revealed on average cell purity above 90%. For suppression assays, sorted MDSCs were added at different ratios to splenocytes (2 × 10^5^ splenocytes/well) stimulated with anti-CD3 (1 μg/ml) and anti-CD28 (2 μg/ml) in flat-bottom 96-well plates in RPMI medium supplemented with 10% FCS, 300 μg/ml L-glutamine, 100 U/ml penicillin, 100 μg/ml streptomycin, 1 mM sodium pyruvate, 1 mM non-essential amino acids and 0.02 mM 2-mercaptoethanol in the presence or absence of additional 40 mM NaCl or 80 mM Mannitol solution in the cultures. After 24 h, ^3^H-thymidine was added and T-cell proliferation was measured after another 18 h of culture as counts per minute (cpm) on a Wallac 1450 Liquid Scintillation Counter. Suppressive capacity of MDSCs isolated from HSD or control diet receiving animals was measured in a similar manner, without adding additional NaCl.

### Human MDSC Isolation and Suppression Assay

PMN-MDSCs and autologous CD3^+^ responder T cells from cancer patients were isolated and tested in suppression assays as described before (28). In brief, MDSCs were isolated from CD3-depleted PBMC by FACS using anti-human CD66b-FITC, anti-human CD33PE, anti-human HLA-DR-APC, and anti-human lineage cocktail (CD3, CD20, CD19, CD56, all BV421). Post sort analysis by FACS revealed a purity of at least 90%. T lymphocytes were labeled with 10 μM Cell Proliferation Dye eFluor® 450 (CPDye405) according to manufacturer instructions (eBioscience, Frankfurt am Main, Germany). For induction of T cell proliferation cells were stimulated in L-arginine free RPMI 1640 medium (Thermo Fisher scientific, Karlsruhe, Germany) supplemented with 10% (v/v) heat-inactivated FCS, 100 IU/ml penicillin, 100 mg/ml streptomycin (Thermo Fisher scientific), and 150 μM L-Arginine (both Sigma-Aldrich) in 96 well round bottom plates coated with CD3 (1 μg/ml, clone OKT-3, eBioscience) and CD28 (2 μg/ml, clone 28.2, Beckman coulter). Autologous PMN-MDSC subsets were added in a T-cell: MDSC ratio of 2.5:1. To study the effect of high salt conditions additional 40 mM NaCl solution (Sigma-Aldrich) were added to the medium. CPDye405 intensity was analyzed by flow cytometry after 4 days of co-culture and proliferation. Proliferation index calculation is based on dye dilution and was calculated with ModFit LT3.3 (Verity Software, Topsham, US) according to an algorithm provided by the software. Written informed consent was obtained from all human subjects prior to inclusion in this project in accordance with the ethical standards of the institutional review board, ethical approval was granted by University of Essen, Germany (07/3500 and 16/7135).

### Immunohistochemistry

Immunohistochemistry on tumor sections was done as described before (26). In brief, 5 μm sections of OCT-tissue tech (Sakura) embedded LLC tumor tissues were mounted on slides air-dried overnight and fixed in acetone for 10 min and air-dried for another 20 min. Slides were treated with 0.2% galantine (Sigma Aldrich) and 0.2% Triton X-100 in PBS and additionally blocked with antibody diluent (Dako) for 1 h at RT. All antibody stainings were performed in Dako antibody diluent solution. Primary antibodies were incubated overnight at 4°C. After 3 times washing with PBS, second antibodies were added for 1 h together with Hoechst 33342 (Sigma Aldrich) at room temperature. Negative controls were generated by staining with secondary antibodies and Hoechst 33342 only. After staining, the slides were covered with slowfade (Life Technologies) and analyzed with ObserverD.1 or LSM710 confocal microscopes (Zeiss). The following anti-mouse antibodies were used for confocal and fluorescence microscopy: CD31 (clone MEC13.3, BD # 550274, isotype control rat IgG2a), cleaved caspase 3 (cell signaling # 9604S), CD146 (clone ME9-F1, BD # 562230, isotype control rat IgG2a). As secondary antibodies Alexa 488-, Alexa 568-, and Alexa 647- labeled: anti-rat IgG and anti-rabbit IgG (Life Technologies) were used. Hoechst 33342 was used for staining nuclei.

### Statistical Analysis

Statistical analysis was performed using GraphPad Prism (GraphPad Software). Data were analyzed by unpaired *t*-test. Data tested against a specified value were analyzed by one-sample *t*-test. Repeated measurement two-way ANOVA using Sidak's multiple comparison tests was applied on tumor growth data. Data were presented, if not indicated elsewhere, as mean ± S.E.M. *P* < 0.05 was considered to be statistically significant (^*^p < 0.05, ^**^p < 0.01, ^***^p < 0.001).

## Results

### High Salt Intake Inhibits Tumor Growth in Mice

**Figure 1:**
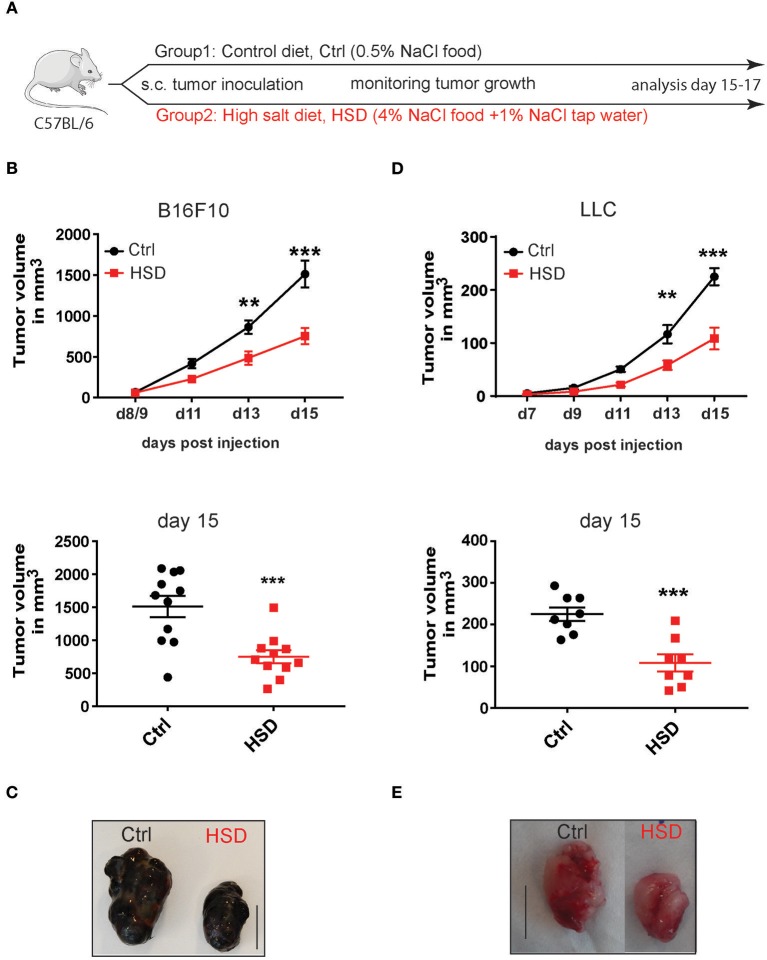
High salt diet inhibits tumor growth in mice. **(A)** Experimental design. C57BL/6 mice were kept on control diet (Ctrl) or were fed a high salt diet (HSD) before tumor inoculation. After tumor challenge the mice were further kept on the same diets until sacrifice. **(B)** Mice pre-fed on the respective diets were challenged with B16F10 melanoma cells by subcutaneous (s.c.) injection. Growth curve and dotplot shows tumor volume as mean ± S.E.M. (*n* = 11) at the indicated time points pooled from two of three independent experiments with similar results. **(C)** Representative pictures were taken from tumors of control and HSD fed mice at day 15 post-induction (p.i.) (scale bar = 1 cm). **(D)** Mice that were fed a control diet or HSD for 2 weeks were subcutaneously injected with Lewis lung carcinoma cells (LLC) and tumor growth was monitored over time. Growth curve and dotplots shows tumor volume as mean ± S.E.M. (*n* = 8) at the indicated time points from one representative of three independent experiments with at least 5 mice per group. **(E)** Representative pictures were taken from LLC tumors of control and HSD fed mice at day 20 p.i. (scale bar = 1 cm). Statistical analysis was performed by Two-way repeated-measure Anova test (^**^p < 0.01, ^***^p < 0.001).

To examine the effects of HSD on cancer development we used the B16F10 syngeneic melanoma transplantation model. This poorly immunogenic tumor model (29, 30) was chosen to analyze possible immune activating effects of a HSD in mice. We first applied a protocol previously used in models of hypertension and autoimmunity by pre-feeding mice with a 4% NaCl containing chow and 1% NaCl in the drinking water compared to a control diet before tumor inoculation (Figure 1A) (1, 4). Of note, HSD fed mice showed a significantly inhibited tumor growth in the B16 tumor model (Figure 1B). Delayed tumor outgrowth was evident as early as day 11 post-injection (p.i.), leading to significant differences in tumor size between both groups at day 13 p.i. and at the day of sacrifice (day 15–17 p.i.) (Figures 1B,C). This effect seemed to be specific for the dietary regimens, since besides water intake no other confounders like e.g., general appearance, weight gain, and food intake were different (Supplementary Figure 1 and data not shown) nor was there a direct effect of high sodium concentrations (additional 40 mM NaCl) on tumor cell viability during *in vitro* culture nor any effect of mannitol as an osmolyte control (Supplementary Figure 2). The NaCl concentrations used *in vitro* are comparable to the *in vivo* situation in high salt fed animals (24, 25). Only at concentrations much higher than 40 mM NaCl high salt conditions were toxic to tumor cells as reported for other tested cell types before (1) (Supplementary Figure 2). To examine if the results were reproducible also in other transplanted tumor models, we tested the HSD regimen in the Lewis lung carcinoma model (LLC) (31). Similar to the B16 model, HSD also significantly delayed LLC tumor growth (Figures 1D,E). Thus, HSD was able to significantly inhibit tumor growth in two independent tumor transplantation models.

### Salt-Induced Changes of the Immune System in Tumor-Bearing Mice

**Figure 2:**
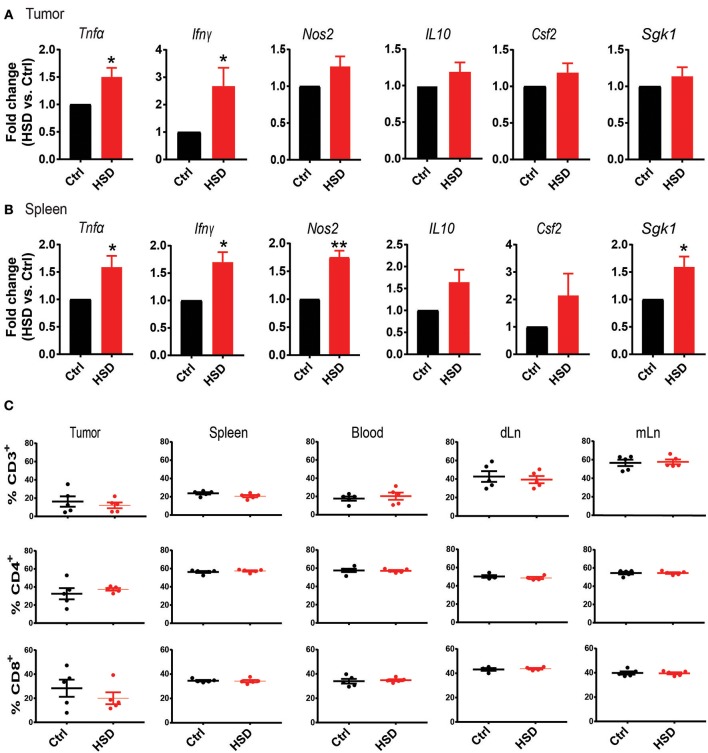
High salt diet creates a pro-inflammatory environment in tumor-bearing mice. **(A)** Quantitative RT-PCR analysis of LLC tumor tissue. Bar graphs show fold change as mean ± S.E.M. from HSD samples normalized to control samples. Data are pooled from two independent experiments (*n* = 8–10). **(B)** Quantitative RT-PCR analysis of spleen samples from LLC tumor-bearing mice. Bar graphs show fold change as mean ± S.E.M. from HSD samples normalized to control samples (*n* = 5). **(C)** Tumors as well as the indicated organs from tumor-bearing mice were subjected to FACS analysis of T cell subsets. Cellular events were defined according to an extended lymphocyte gate, excluding doublets and dead cells. T cells were defined as CD3^+^ and further gated according to CD4 and CD8 expression. Bar graphs show mean ± S.E.M. of CD3^+^ cells (upper row), CD4^+^ T cells (center row, gated on CD3^+^), and CD8^+^ T cells (lower row, gated on CD3^+^). Samples were analyzed on day 15–17 p.i. statistical significance was determined by *t*-test (^*^p < 0.05, ^**^p < 0.01).

Since it is well-known that a HSD could have a profound impact on the host immune system by several mechanisms (7) we first analyzed general immune parameters in tumor-bearing mice receiving a HSD compared to controls. Transcriptional analysis by quantitative real-time PCR with reverse transcription (qRT-PCR) of tumor tissue from mice at day 15–17 after tumor cell inoculation revealed a significant increase of tumor necrosis factor alpha (*Tnf*α), interferon-γ (*Ifn*γ) and a tendency of increased nitric oxide synthase 2 (*Nos2*) expression (*p* = 0.0549), whereas transcripts for interleukin (IL) 10 (*Il10*) and granulocyte macrophage-colony stimulating factor 2 (*Csf2*) remained unchanged (Figure 2A). The expression of serum and glucocorticoid-regulated kinase (*Sgk1)*, as a prominent salt signature gene (32) was similarly not changed (Figure 2A). When analyzing spleen cells from tumor-bearing mice at day 15–17 p.i., we detected similarly significant changes in *Tnf*α, *Ifn*γ, and *Nos2* expression and a significant increase in *Sgk1* expression (Figure 2B). Similar to the LLC model, we found increases in *Tnf*α, *Ifn*γ, and *Nos2* expression in the HSD B16 model (Supplementary Figure 3A). However, in both tissues we were unable to detect changes in *Il17a* expression. Moreover, ELISA-mediated analysis of serum cytokines of tumor-bearing animals on day 15–17 p.i. did not show any differences for TNFα, IFNγ, IL-10, or IL-17A between both groups (data not shown). However, by intracellular FACS analysis we detected increases of TNFα and IFNγ expression in tumor infiltrating cells (Supplementary Figure 3B).

We next analyzed by FACS the abundance of CD3^+^, CD4^+^, and CD8^+^ T cells in different tissues of tumor-bearing mice. FACS analysis of tumors, spleens, peripheral blood, tumor draining lymph nodes (dLN) and mesenteric lymph nodes (mLN) revealed no significant changes between control and HSD groups (Figure 2C; Supplementary Figure 3C). Although there were no obvious changes in T cell populations, particularly the higher expression of *Tnf*α and *Ifn*γ indicated a more pro-inflammatory environment in HSD fed mice compared to controls and suggests that the observed effect of delayed tumor growth is potentially related to changes in the host immune system.

### High Salt Mediated Effect on Tumor Growth Is Largely Independent of T Cells

**Figure 3:**
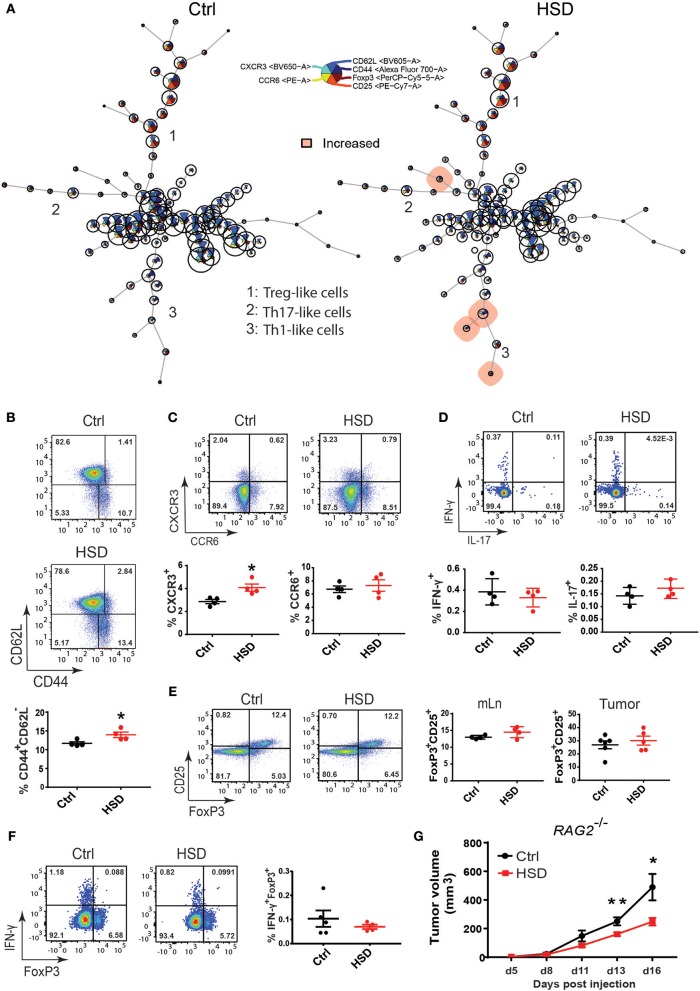
The impact of T cells on high salt mediated reduced tumor growth. **(A)** FlowSOM visualization of flow cytometry data across mesenteric lymph nodes (mLN). Single live CD4^+^ cells for each sample (Ctrl, *n* = 4; HSD, *n* = 4) were exported and concatenated then analyzed using FlowSOM, which arranges the cells into clusters (represented by circles) according to similarities in their expression profiles. Each node represents one cluster (total = 100 nodes). Colored nodes highlight statistically significant changes (*p* < 0.05) in cell population between two groups (HSD and Ctrl). **(B)** The same mLN samples as in **(A)** were analyzed by manual gating for **(B)** CD4^**+**^ effector-memory T cells (CD44^**+**^CD62L^**−**^). **(C)** Th1-like cells (CD4^**+**^CXCR3^**+**^) and Th17-like cells (CD4^**+**^CCR6^**+**^). **(D)** IL-17 and IFNγ producing CD4^**+**^ T cells after PMA/Ionomycin restimulation and intracellular staining. Statistical significance was determined by unpaired *t*-test (^*^p < 0.05). **(E)** FoxP3^**+**^CD25^**+**^ regulatory T cells in tumor-bearing mice. FACS plots show the indicated parameters after pre-gating on CD3^**+**^CD4^**+**^ T cells. Dotplots show frequency of the indicated populations as mean ± S.E.M. (*n* = 4/group) representative of three independent experiments. **(F)** IFNγ-producing regulatory T cells after restimulation of mLN single cell suspensions from LLC tumor-bearing mice. Representative FACS plots show FoxP3 against IFNγ after pre-gating on CD3^**+**^CD4^**+**^ T cells. Dotplots show frequency of FoxP3^**+**^IFNγ^**+**^ cells as mean ± S.E.M. from 4 to 5 mice in each group. Similar results were obtained from B16 tumor-bearing mice. **(G)** *RAG2*^**−/−**^ mice were fed a high salt diet (HSD) or control diet (Ctrl) and challenged with LLC tumor cells. Growth curve shows tumor volume as mean ± S.E.M. for 7 mice in each group. Statistical analysis was performed by Two-way repeated-measure Anova test (^*^p < 0.05, ^**^p < 0.01).

Since it is known that a HSD can profoundly affect the phenotype and function of CD4^+^ T cells, particularly T_H_17 cells and Tregs (1, 4, 6, 33, 34), we further examined these subsets in more detail in tumor-bearing animals receiving either a HSD or control diet by multicolor FACS analysis of different tissues as shown in Figure 2C. In line with the increased cytokine expression (Figures 2A,B; Supplementary Figure 3A), we detected a significantly higher number of effector-memory CD4^+^ T cells (T_EM_) and T_H_1-like cells in the mLN of HSD fed tumor-bearing mice based on an antibody panel containing CD3, CD4, CD44, CD62L, CCR6, and CXCR3 specific antibodies by FlowSOM analysis at day 15–17 p.i. (Figure 3A). However, cells isolated from other tissues, including tumor-infiltrating cells, did not show any significant differences (Supplementary Figure 4 and data not shown). The higher percentage of T_EM_ cells was confirmed by a manual gating strategy for CD44 and CD62L expression in CD4^+^ T cells of mLN (Figure 3B). HSD fed animals further displayed a higher percentage of CXCR3^+^ CD4^+^ T cells in mLN cells, indicative of an increase in T_H_1 cells in HSD fed tumor-bearing animals (Figure 3C). Of note, an increase of T_H_1 cells was reported before in a model of lupus nephritis in an SGK1 dependent manner (35) and possibly could explain the HSD mediated effect on tumor growth. However, we only detected subtle changes of *Sgk1* expression (Figures 2A,B) that makes this scenario unlikely. Moreover, there seemed to be no changes in T_H_17-like cells based on CCR6 chemokine receptor expression (Figure 3C).

We next extended the analysis by intracellular FACS after PMA/ionomycin restimulation *in vitro* for intracellular cytokine detection. Again, we were not able to detect significant changes for IL-17A (Figure 3D), indicating that T_H_17 cells may not play a significant role in the delayed tumor growth of animals receiving a HSD. In contrast to the observed increases of T_H_1-like cells, we were also not able to detect more IFNγ expressing cells in HSD fed mice, indicating that the observed increase of T_H_1-like cells based on CXCR3 expression in mLN is of rather minor relevance (Figure 3D). This was similar in all tissues analyzed (data not shown).

Since we and others demonstrated before that a HSD could also impact Foxp3^+^ Tregs (7, 34), for instance by inducing a T_H_1-like effector phenotype, we carefully analyzed the frequency and cytokine expression of Tregs in different tissues of both groups. However, the detailed analysis of Foxp3^+^ Tregs did not show any significantly altered frequency or phenotype (Figures 3E,F; Supplementary Figure 5) indicating that Tregs may not play a critical role in the HSD induced delayed tumor growth in this model. Besides CD4^+^ T cells, we were not able to detect any significant changes in CD8^+^ T cells nor NK cells in the tissues and at time points analyzed (Supplementary Figures 6, 7).

Having analyzed changes in the phenotype and function of T cells in high salt fed tumor-bearing mice, we next wanted to directly evaluate the contribution of T cells to tumor growth reduction mediated by HSD. To this end, we applied a similar tumor transplantation model using LLC and B16 tumor cells in *RAG2* deficient animals, completely lacking mature T and B cells (36). Surprisingly, while tumor growth reduction was slightly less efficient, the HSD effect was still detectable in *RAG2* deficient mice (Figure 3G; Supplementary Figure 8), indicating that T cells rather play a minor role in this model. Thus, although we were able to detect differences in T cell populations between both groups, the HSD mediated effect on tumor growth seemed to be mostly independent of alterations in T cells as well as B cells.

### High Salt Intake Modulates Myeloid-Derived Suppressor Cells

Since T cells were not the major driving force behind the HSD mediated effect on tumor growth, we further analyzed innate immune cells in more detail. We and others have shown before that particularly M1-type- and M2-type macrophages were sensitive to HSD conditions (2, 3, 37). Thus, we hypothesized that innate immune cells may be key for the observed effect of inhibited tumor growth in HSD receiving animals. In this respect, particularly myeloid-derived suppressor cells are known to have a critical impact on tumor growth (17, 28). MDSCs are classified as myeloid cells with suppressive function and can be identified in mice using the markers CD11b, Ly6C, and Ly6G for PMN-MDSC (CD11b^+^Ly6G^high^Ly6C^low^) and M-MDSCs (CD11b^+^Ly6G^−^Ly6C^high^) (17, 28, 38–40).

**Figure 4:**
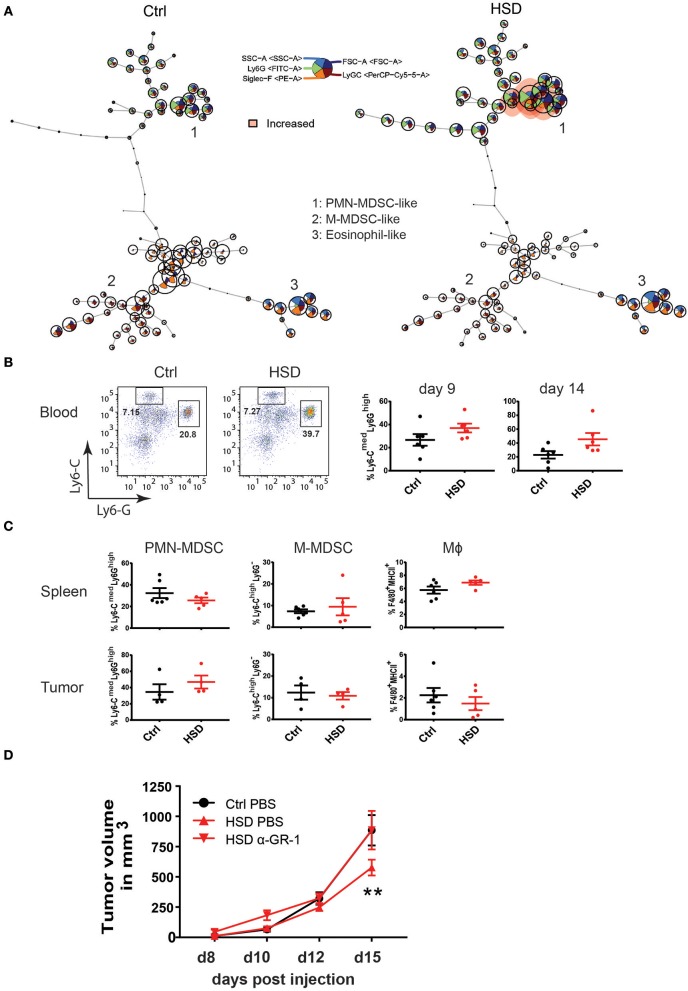
High salt diet mediates changes in myeloid cells in tumor-bearing mice. **(A)** FlowSOM visualization of flow cytometry data in blood. Single live CD45^+^CD11b^+^ cells for each sample (Ctrl and HSD, *n* = 6/group) were exported and concatenated then analyzed using FlowSOM, which arranges the cells into clusters (represented by circles) according to similarities in their expression profiles. Each node represents one cluster (total = 100 nodes). Colored nodes highlight statistically significant changes (*p* < 0.05) in cell population between two groups (HSD and Ctrl). **(B)** Blood samples from B16 tumor-bearing mice were analyzed for MDSC populations at day 9 and 14 p.i. FACS plots show representative distribution of M-MDSC-like cells (Ly6-C^high^LyG^**−**^ and PMN-MDSC-like cells (Ly6C^med^Ly6-G^high^) after gating on CD11b^+^ cells. Bar graphs show the frequency of each population in high salt diet fed mice (HSD) compared to control mice (Ctrl) as mean ± S.E.M. from 6 mice in each group. Statistical significance was determined by unpaired *t*-test. **(C)** Single cell suspensions from spleens and tumors were analyzed as in **(B)** at day 16. Dotplots show the frequency of each population in high salt diet fed mice (HSD) compared to control mice (Ctrl) as mean ± S.E.M. from 5 to 6 mice in each group. **(D)** High salt diet (HSD) fed mice were treated with an anti-GR-1 antibody or PBS as control from day 4 after B16 melanoma cell inoculation on consecutively every second day. Growth curve shows tumor volume as mean ± S.E.M. of 5 mice in each group. Statistical analysis was performed by Two-way repeated-measure Anova (^**^p < 0.01).

We examined subsets of myeloid cells including potential MDSC populations in tumor-bearing mice receiving either HSD or control diet by FACS in different tissues at different time points (Figure 4). Of note, FlowSOM analysis of samples from peripheral blood at day 14 p.i. showed significant changes in myeloid cell populations (Figure 4A). We observed a similar trend in CD11b^+^Ly6G^high^Ly6C^low^ PMN-MDSCs by manual gating (Figure 4B; Supplementary Figure 9) and confirmed an increase of PMN-MDSCs at day 14 p.i. (*p* = 0.0559) as well as day 9 (*p* = 0.1336), although not reaching statistical significance. When analyzing MDSC subsets in tumors and spleens at day 15–17 p.i., however, we could not detect any significant differences in frequencies of MDSCs (Figure 4C; Supplementary Figure 10). In addition, we could not reveal any significant changes in monocyte/macrophage populations (Figure 4C; Supplementary Figure 10), indicating that by HSD the composition of myeloid cell populations was changed systemically (in peripheral blood) but not locally in the tumor. Since MDSCs could also affect tumor angiogenesis (15), we analyzed abundance of endothelial cells in tumor sections by CD31 immunohistology. Specificity for blood vessel endothelial cells was confirmed by CD146 co-staining (41) (data not shown). However, we could not detect major differences in CD31 staining indicative for alterations in the degree of tumor angiogenesis between the two groups at time points analyzed (Supplementary Figure 11).

The above data suggested that HSD induced changes on the myeloid compartment and that particularly MDSCs might be key to HSD induced inhibited tumor growth. To directly test the impact of MDSCs in the HSD tumor model, we applied an antibody-mediated depletion of MDSCs by using anti-GR1 antibodies, known to be very efficient in depleting MDSCs in mice (42). In line with previous studies (43), this protocol efficiently depleted MDSCs and neutrophils in the model system as monitored by FACS analysis in blood and tumors (Supplementary Figure 12). Importantly, by depleting MDSCs using anti-GR1 antibodies, the inhibitory effect of HSD on tumor growth was completely abolished (Figure 4D). Antibody treated HSD fed animals displayed a similar tumor growth as animals receiving the control diet. These data indicate that the population of MDSCs are essential players in the HSD mediated inhibitory effect on tumor growth.

### High Salt Blocks Suppressive Function of Murine and Human MDSCs

**Figure 5:**
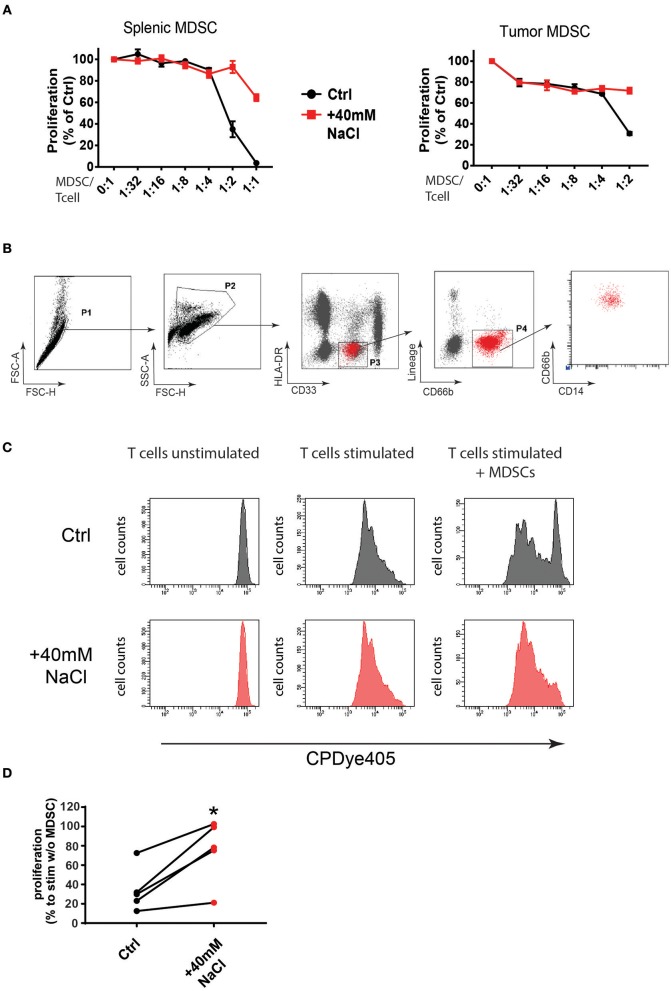
High salt conditions block murine and human MDSC function *in vitro*. **(A)** MDSCs isolated from LLC tumors and spleens of tumor-bearing mice were cultured with splenocytes at the indicated ratios in the presence of anti-CD3/CD28 stimulating antibodies. Cells were either cultured under high salt (+40 mM NaCl) or control conditions (Ctrl). Proliferation of responder splenocytes was measured by ^3^H-thymidin incorporation. Curves show proliferation normalized to controls (stimulated splenocytes without addition of MDSCs) as mean ± S.E.M from triplicates representative for three independent experiments with similar results. **(B)** PMN-MDSCs were sorted from PBMCs of patients with oropharynx or bladder cancer. FACS plots show the gating strategy. **(C)** FACS purified human PMN-MDSC were co-cultured with CPDye405-labeled autologous CD3^+^ T cells under control (Ctrl) or high salt conditions (+40 mM NaCl) for 4 days at a 1:2.5 ratio. FACS histograms from a representative patient are shown. **(D)** Graph shows proliferation index from five independent patients under control or high salt (+40 mM NaCl) conditions. Statistical significance was determined by unpaired *t*-test (^*^p < 0.05).

The above data clearly pointed toward phenotypic and functional changes of MDSCs upon HSD and indicated that these changes in MDSC populations significantly contributed to the inhibitory effect of high salt on tumor growth. Although the number of MDSCs in spleen and tumor was not altered upon HSD, it is possible that HSD alters the function of these cells. To further test if high salt may directly affect MDSCs, we analyzed the impact of increased sodium concentrations on MDSC phenotype and function *in vitro* (Figure 5). To mimic *in vitro* the high salt conditions in the interstitial tissues of animals receiving a HSD diet (24, 25), we used an established protocol by increasing the sodium concentration in cell cultures by adding an additional 40 mM of NaCl (1, 3, 4, 33, 34). To test the effects of high salt on MDSC function we isolated MDSCs from spleens and tumors of LLC tumor-bearing mice and examined these cells *in vitro* for their capacity to suppress effector T cells under control or high salt conditions (+40 mM NaCl). It has been shown that immature myeloid cells can affect tumor growth also in the absence of T cells (19) and although the *in vivo* experiments in *RAG2* deficient mice clearly pointed to a T cell independent mechanism for myeloid cell mediated tumor growth reduction, we still resorted to the *in vitro* T cell suppression assay, as an established method to analyze changes in immunoregulatory phenotype after salt exposition.

MDSCs were isolated based on the expression of specific markers as described before (16, 21) (Supplementary Figures 13A,B). Of note, high salt conditions blocked MDSC suppressive capacity *in vitro* almost completely (Figure 5A; Supplementary Figure 14A). Particularly, the effect was observed in M-MDSCs isolated from tumors and spleens indicating that this subset was highly affected by high sodium conditions *in vitro*. A similar tendency, although to a lower extent, was also observed for PMN-MDSCs (data not shown). As an osmolyte control, we tested the effects of 80 mM mannitol in cultures but did not observe a similar effect, indicating that the effect was rather specific to Na^+^ ions and was not simply due to changes in osmotic pressure (Supplementary Figure 13C). Of note, a similar effect of functional inactivation was also observed, when testing the suppressive capacity of MDSCs isolated from HSD and control fed mice. MDSCs isolated from tumors of HSD fed mice showed lower suppressive function compared to MDSCs isolated from tumors of control fed mice, indicating that the functional changes occur also under high salt conditions *in vivo* (Supplementary Figure 14B).

To examine if this effect may also apply to human MDSCs, we isolated MDSCs from peripheral blood of cancer patients (Supplementary Table 1). Since in humans the subset of PMN-MDSCs have been demonstrated to be the subset with highest suppressive activity and clinical relevance in cancer patients (16, 28), we isolated MDSCs as HLA-DR^−^CD33^dim^CD66b^+^Lin^−^CD14^dim^ cells from five independent cancer patients (Figure 5B). We then tested MDSC functionality in suppression assays under control or high salt (+40 mM NaCl) conditions as measured by fluorescent dye dilution of labeled T cells as described before (28) (Figure 5C). Of note, high salt conditions blocked the suppressive function of MDSCs in T cell suppression assays of all five patients (Figure 5D). This data clearly show that increased sodium concentrations, mimicking high salt conditions in tissues of HSD fed animals, could indeed markedly change the inflammatory phenotype of MDSCs and alter their function (here shown for immunosuppression) in mice and humans. While it remains to be shown how exactly myeloid cells reduce tumor growth, the animal experiments using HSD show that such changes can directly affect tumor growth *in vivo*.

## Discussion

High dietary salt intake is believed to be associated with various diseases (5). Besides implications in cardiovascular pathologies, recent data have clearly shown that high salt intake could profoundly modulate the immune system through direct and indirect mechanisms-mainly leading to shifts toward a pro-inflammatory milieu (6, 7). However, the majority of current *in vivo* data is based on studies in rodents, using protocols of extremes of high salt intake that likely cannot extrapolate to humans and therefore findings have to be analyzed carefully if they can apply to the human situation (7, 44). Nevertheless, few available studies in humans indicate that even moderate changes in salt intake could impact host immunity and clinical parameters in a similar manner compared to experimental animal studies. For instance, a daily increase of 6 g NaCl for 14 days seemed to affect already T_H_17 cell frequency and blood pressure as reported for a small human pilot study (4, 45). The data presented here indicates that a high salt diet could also strongly affect tumor growth by enhancing anti-tumor immunity through the modulation of MDSC function. Thus, in the context of an immune response to cancer, a high salt diet may positively affect anti-tumor immunity, similar to enhanced immune responses toward certain pathogens as shown before for *Leishmania* infection in skin (2).

In humans, high sodium intake might be a risk factor for the development of gastric cancer (46, 47). However, animal studies on gastric cancer development show contradictory results (48–51) indicating that the exact role of sodium intake in cancer development is still not well-defined and the stomach represents a very peculiar milieu because of the acidic nature and the ionic composition in the gastric mucosa.

When analyzing major immune parameters in tumor-bearing mice between control and high salt fed mice, we could detect increases in *Tnfa, Ifng*, and *Nos2* expression in spleen and tumor tissues, indicating a more pro-inflammatory environment in HSD mice. Although we couldn't measure any significant changes in T cell frequencies between the two groups, HSD impacted adaptive immune cells in our model, as measured by increased effector-memory and T_H_1-like cells in tumor-bearing mice. However, this was only evident in mLN of tumor-bearing mice, indicating that these changes may not play a relevant role for the observed effects. A possible explanation for this observation might be the known impact of HSD on the gut microbiota and T cells (4). We neither did detect changes in T_H_17 cells, a T cell subpopulation usually enhanced under high salt conditions particularly in experimental settings of neuro- and gut-inflammation. However, this can be due to the nature of the inflammatory conditions of the tumor models used or due to different time points and tissues analyzed compared to other experimental models of inflammation under HSD (45). In contrast to CD4^+^ T effector cells, we were not able to detect any significant differences in the CD8^+^ T cell compartment at the examined time points in different tissues. Since regulatory T cells can also critically be affected by high salt (34) and they greatly impact anti-tumor immunity (52), we also examined this suppressive CD4^+^ T cell subset thoroughly in our model. However, Foxp3^+^ Tregs didn't show any differences in frequency or altered subpopulations [e.g., IFNγ^+^ T_H_1-like Tregs (34)] between both groups in all tissues analyzed. Nevertheless, we cannot exclude functional changes, since we did not test the *in vitro* suppressive capacity of isolated Tregs from tumor-bearing mice that was shown to be impaired under high salt conditions *in vitro* and in humanized mouse models in settings of a xenogeneic graft-vs.-host disease model (x-GvHD) *in vivo* (34). However, importantly, since we observed almost similar effects of delayed tumor growth under HSD in *RAG2*^−/^^**−**^ animals, the effect seems not to be critically dependent on T cells in both tumor models tested, suggesting that the changes in phenotype seen in the T cell compartment are a reflection of the changes in the pro-inflammatory milieu but do not significantly contribute to tumor control.

Therefore, we further examined innate immune cells in tumor-bearing mice. Monocyte/macrophages have been shown before to be sensitive to high salt (2, 3, 37) and particularly M1-type macrophages were induced to become more pro-inflammatory in an p38MAPK/NFAT5 dependent manner and to express higher levels of a pro-inflammatory gene signature, including *Tnf*α and *Nos2* (2, 37) two genes that were also highly induced in HSD fed animals. However, we could not detect any significant changes in frequencies of monocyte/macrophage populations locally in the tumor milieu, although we cannot exclude functional changes on these cells upon HSD. In addition, we could not detect any changes in NK cell frequencies. Nevertheless, when analyzing CD11b^+^Ly6G^high^Ly6C^low^ and CD11b^+^Ly6G^−^Ly6C^high^ myeloid cell populations in tumor-bearing animals that include MDSCs, we observed systemically increased numbers of CD11b^+^Ly6G^high^Ly6C^low^ MDSC-like cells in high salt fed mice. Particularly in blood at early time points after tumor inoculation, these PMN-MDSCs seemed to be increased in cell numbers under HSD compared to controls, indicating an effect of HSD in tumor-bearing mice in this myeloid cell population. Since MDSCs are critical modulators of anti-tumor immunity (16–18) we thus analyzed this subset in more detail.

Interestingly, the depletion of GR1^+^ cells annihilated the HSD effect on tumor growth, clearly pointing toward a functional role of MDSCs. We thus tested the impact of high sodium concentrations on the suppressive capacity of MDSCs *in vitro*, as a surrogate marker for their suppressive vs. pro-inflammatory activity. Of note, high salt concentrations *in vitro*, mimicking sodium content in tissues of HSD fed animals, significantly blocked the function of MDSCs isolated from tumor-bearing mice in suppression assays. Moreover, MDSCs isolated directly from tumors of HSD fed mice showed similarly a lack of suppression compared to cells isolated from tumors of control fed mice. In this respect, it is of interest that a previous study found accumulation of functional MDSCs in different models of hypertension, including a salt sensitive setting, implicating a functional role in blood pressure regulation. However, how the specific salt sensitive L-N^G^-Nitroarginine Methyl Ester (L-NAME) model for hypertension compares to the tumor models is currently unclear and would be of interest to be investigated in further studies (53). Our data indicate that high sodium content may directly affect particularly MDSC function in tumor-bearing mice, consequently leading to a shift in the immune balance toward a pro-inflammatory environment. However, since T cells do not seem to be the major force driving the anti-tumoral effect, as demonstrated by the *RAG2*^−/^^**−**^ experiments, the key executing cell type seems to be rather of innate origin. Possibly, as observed by increases of *Tnfa, Ifng*, and *Nos2* expression, it could well be that in HSD fed animals besides other innate immune cells like macrophages, tumor-infiltrating MDSCs themselves became more pro-inflammatory and anti-tumoral. In this respect it is of interest that MDSCs were shown to be highly plastic cell types with the ability to convert toward proinflammatory effector cells (22).

Importantly, a clear modulation of suppressive activity under high salt conditions was also observed in circulating human MDSCs from cancer patients. This indicates that high salt could similarly affect MDSC function in humans. Thus, potential molecular changes induced by high salt conditions may offer novel therapeutic targets to possibly assist cancer immunotherapy. MDSCs are considered to be a major hurdle in cancer immunotherapy, preventing efficient immune attack against tumors e.g., when using checkpoint inhibitors (20, 54). Therefore, the further exploration of this effect may have potential in immune therapies for targeting MDSC function. However, the detailed analysis of the molecular effect has to be addressed in future studies and is out of the scope of this study. It would be of interest for future studies to analyze the role of known molecular high salt targets like p38/MAPK, SGK1 and NFAT5 and if they are also involved in salt exposed MDSCs, as it has been shown for T cell and monocyte/macrophage populations. Of note, p38/MAPK signaling is also of crucial importance for the pro-inflammatory and pro-tumoral activity of tumor-associated PMN in humans (55). In addition, it would be of interest to test in future studies if the functional changes in MDSCs under high salt exposure are also related to epigenetic remodeling and changes in immuno-metabolism as observed for M2-type macrophages (3).

In summary, we show that a high salt diet significantly delays tumor growth in two independent murine tumor transplantation models. This effect seems to be mediated through enhanced anti-tumor immunity by a functional inactivation of MDSCs. Since high salt conditions also affected human MDSCs in a similar manner, our data suggest that the targeting of this mechanism could potentially be a novel beneficial strategy to block MDSC function in settings of cancer immunotherapy.

## Ethics Statement

**Animal studies:**

The protocol was approved by the ethics committees of animal studies at the University of Hasselt (201738) and Vrije Universiteit Brussel (14-220-26).

**Human specimen:**

Written informed consent was obtained from all human subjects prior to inclusion in this project in accordance with the ethical standards of the institutional review board and with the Declaration of Helsinki, ethical approval was granted by University of Duisburg-Essen, Germany (07/3500 and 16/7135).

## Author Contributions

RW and IH designed and performed experiments and analyzed data. LV, MKi, KB, AG, DS, BC-R, LM, EL, DL, and JK performed experiments and analyzed data. TK, SB, and JV supervised experiments and analysis. MKl conceived the project, designed, and supervised experiments and analysis and wrote the manuscript with further input from all authors.

### Conflict of Interest Statement

The authors declare that the research was conducted in the absence of any commercial or financial relationships that could be construed as a potential conflict of interest.
