# Supplementary material for: High Salt Inhibits Tumor Growth by Enhancing Anti-tumor Immunity
Source: Front Immunol. 2019 Jun 4;10:1141. doi: 10.3389/fimmu.2019.01141 (PMC6557976; doi:10.3389/fimmu.2019.01141)
Supplement: Supplementary file 1 [file Data_Sheet_1.pdf]

Supplementary Figure 1

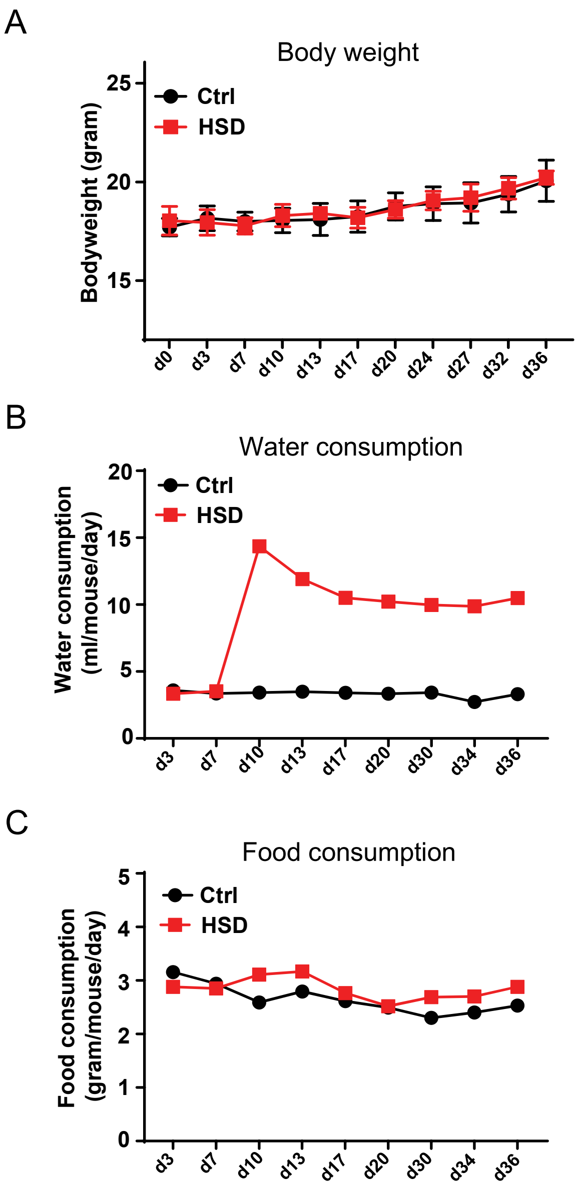

Supplementary Figure 1. Characterisation of tumor-bearing mice. Body weight, water and food consumption was measured over the time course of each experiment for control group (Ctrl) and high salt diet fed mice (HSD). A) Curve shows body weight (gram) as mean ± S.E.M. from 8 mice challenged with LLC tumors. B) Curve shows water consumption as ml/mouse/day at each time point calculated from 8 mice/cage in each group. C) Curve shows food consumption as gram/mouse/day calculated from 8 mice/cage in each group.

B) Curve shows water consumption as ml/mouse/day at each time point calculated from 8 mice/cage in each group. C) Curve shows food consumption as gram/mouse/day calculated from 8 mice/cage in each group.

Supplementary Figure 2

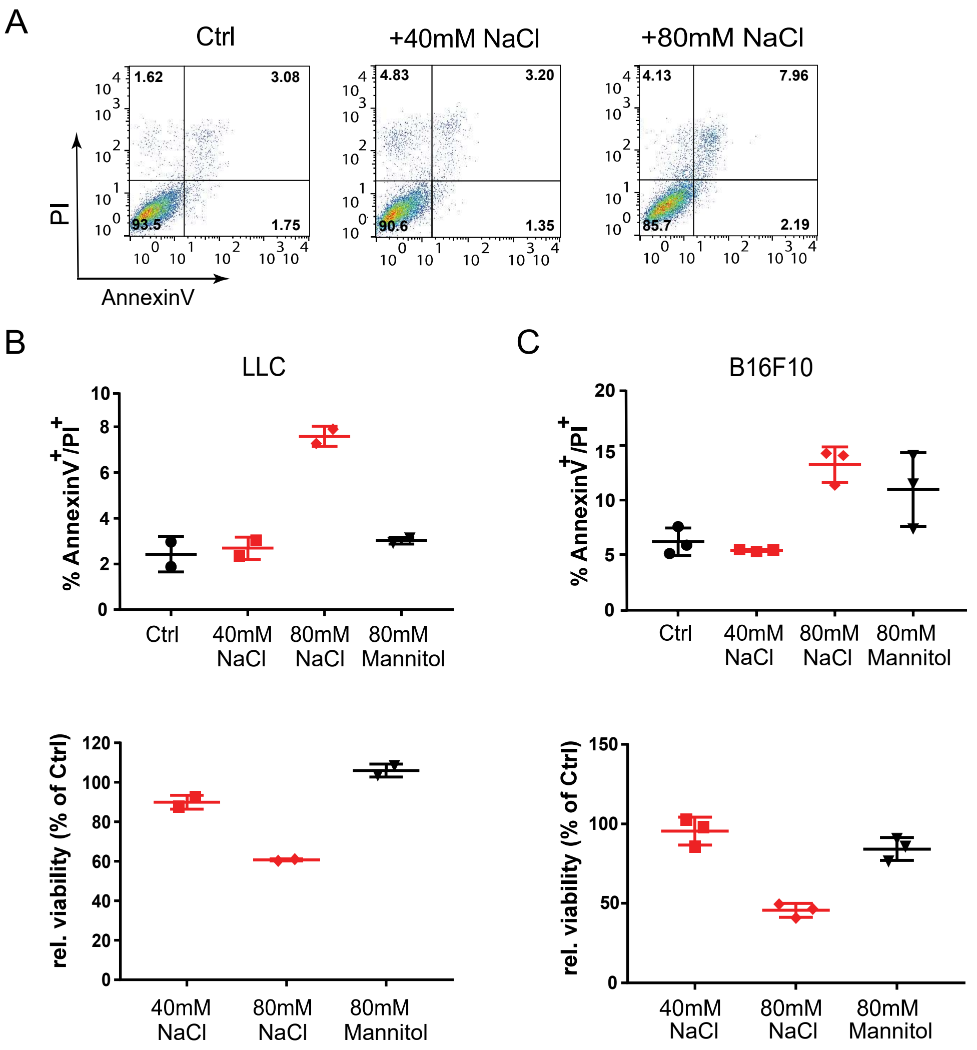

Supplementary Figure 2. Effects of high salt conditions on tumor cell growth *in vitro*. LLC & B16F10 cells were cultured under high salt conditions (+40mM NaCl), +80mM Mannitol as an osmolyte control or were left untreated (Ctrl). After 72 hours cells were harvested and subjected to AnnexinV and propidium iodide (PI) staining to detect apoptotic cells. A) FACS plots show representative stainings for the indicated conditions. B & C) Dotplots show the frequency of AnnexinV$^+$PI$^+$ LLC (B) or B16F10 (C) cells (upper row) and relative viability (lower row, number of viable cells normalized to controls as mean ± S.D. LLC n=2 & B16F10 n=3).

Supplementary Figure 3

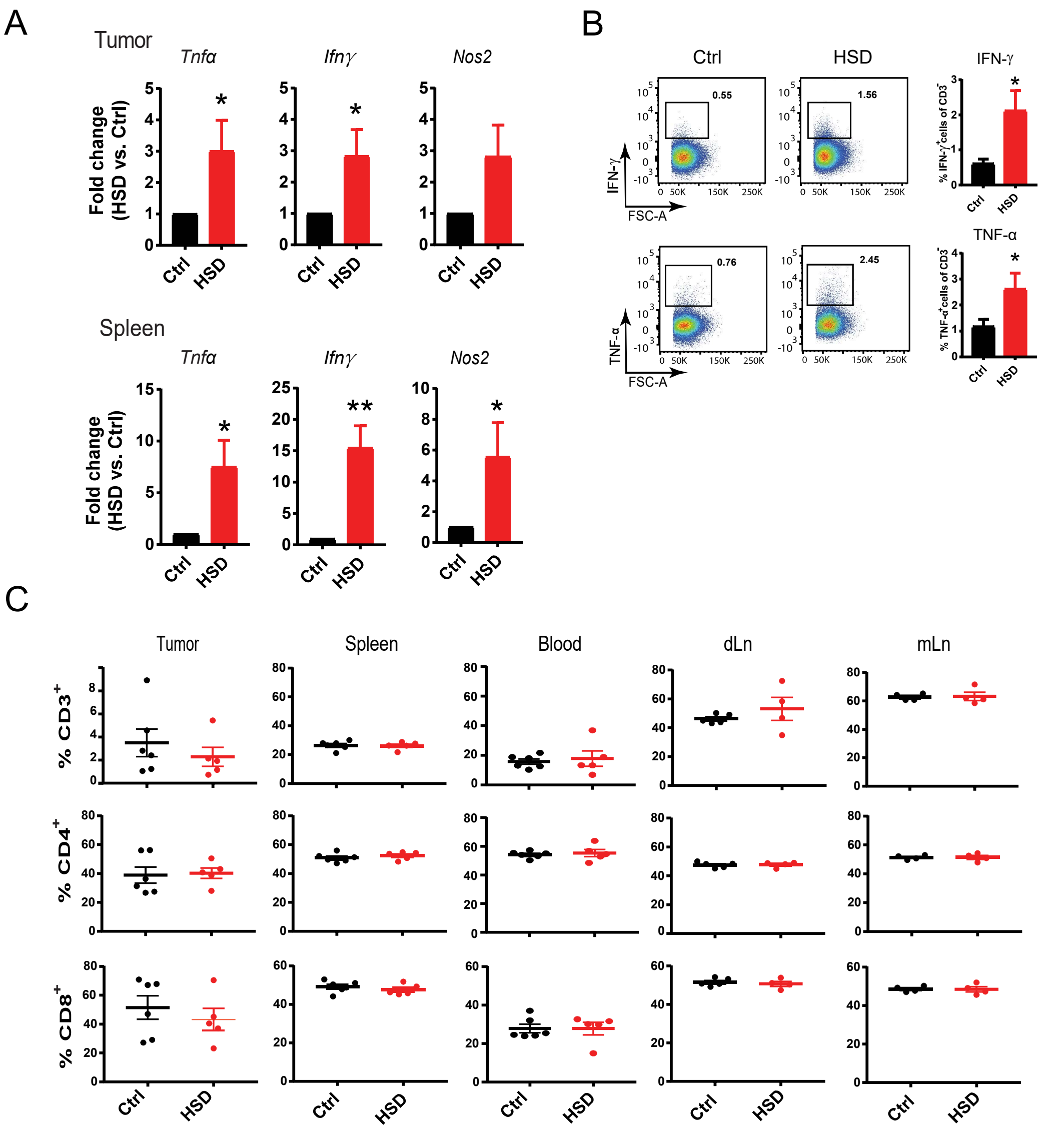

Supplementary Figure 3. High salt diet creates a pro-inflammatory environment in tumor-bearing mice. A) Quantitative RT-PCR analysis of B16 tumor tissue (upper row) or spleens (lower row) on day 16 p.i.. Bar graphs show fold change as mean ± S.E.M. from HSD samples normalized to control samples. Data are pooled from three independent experiments (n=14-16). Statistical significance was determined by t-test (*p<0.05, **p<0.01). B) Dotplots show representative intracellular TNFα & IFNγ staining of $CD3^-$ cells after PMA/ionomycin restimulation.

Bar graphs show frequency as mean ± S.E.M. from 8-10 mice per group. Data is pooled from two independent experiments in the B16 tumor model. Statistical significance was determined by t-test (*$p<0.05$). C) Indicated organs from tumor-bearing mice were subjected to FACS analysis for T cell subsets on day 16. Bar graphs show mean ± S.E.M. of $CD3^+$ cells (upper row), $CD4^+$ T cells (center row, gated on $CD3^+$) and $CD8^+$ T cells (lower row, gated on $CD3^+$).

Supplementary Figure 4

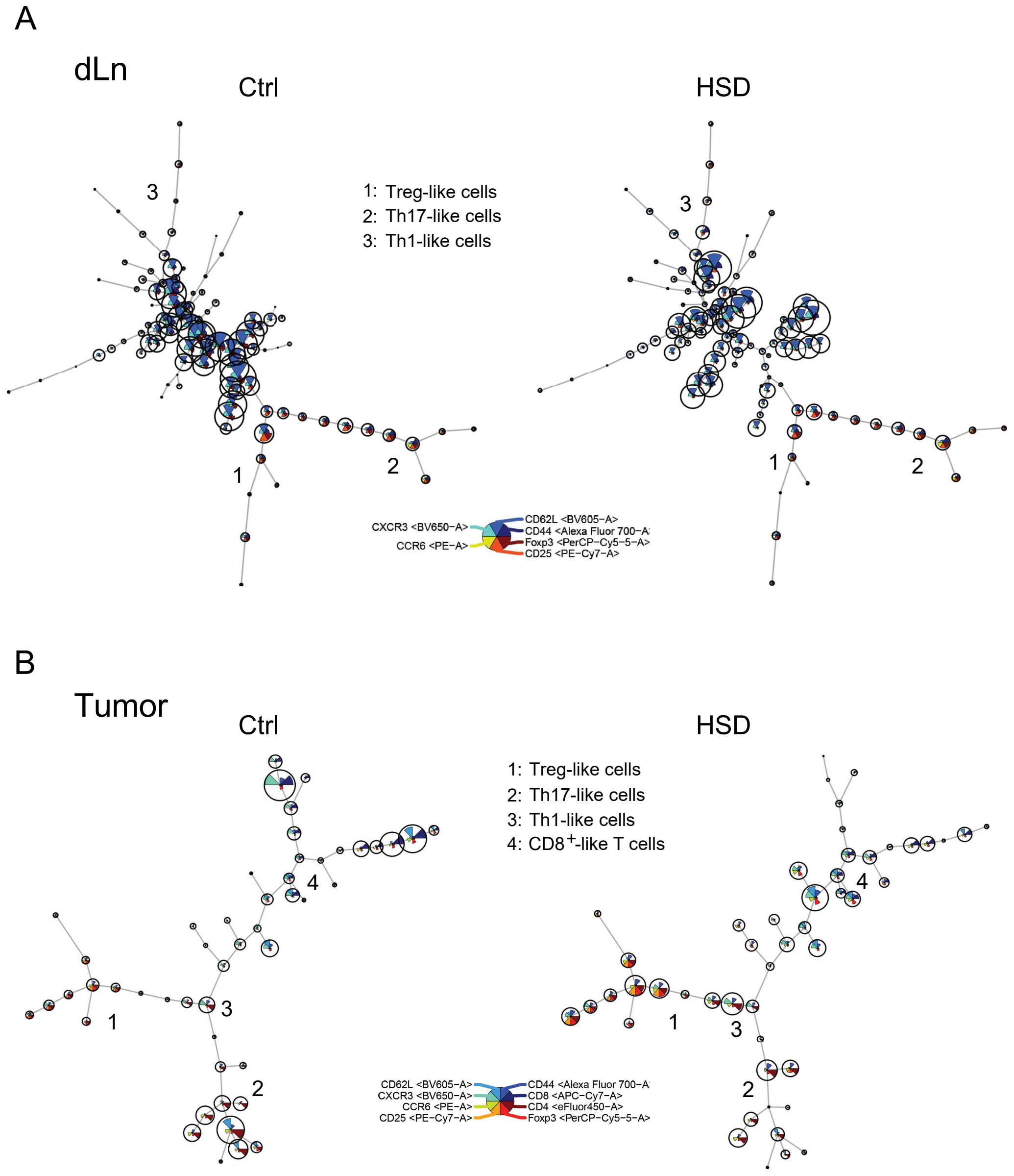

Supplementary Figure 4. Characterisation of CD4^{+} T cells in tumor-bearing mice. A) FlowSOM visualization of flow cytometry data across draining lymph nodes (dLN) of B16 tumor-bearing mice. Single live CD4^{+} cells for each sample (Ctrl and HSD, n=4/group) were exported and concatenated then analyzed using FlowSOM, which arranges the cells into clusters (represented by circles) according to similarities in their expression profiles.

Each node represents one cluster (total = 100 nodes). B) FlowSOM visualization of flow cytometry data across LLC tumor tissue. Single live $CD3^+$ cells for each sample (Ctrl and HSD, n=4/group) were exported, concatenated and then analyzed using FlowSOM which arranges the cells into clusters (represented by circles) according to similarities in their expression profiles. Each node represents one cluster (total = 100 nodes).

Supplementary Figure 5

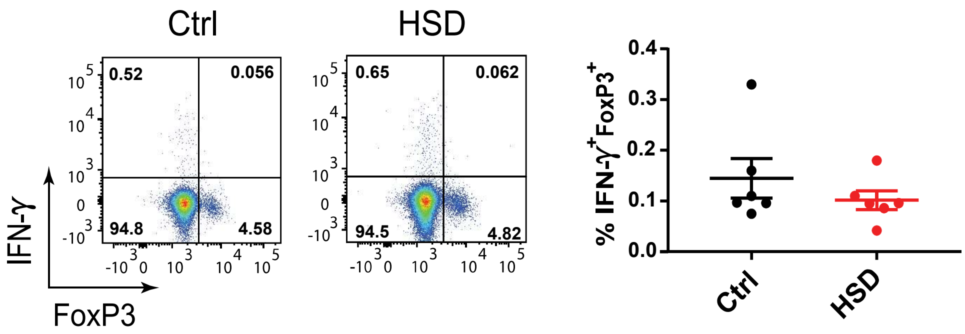

Supplementary Figure 5. Analysis of IFNγ-producing regulatory T cells in B16 tumor-bearing mice. As shown for the LLC model (Figure 3f), IFNγ production was analyzed of regulatory T cells after restimulation of mLN single cell suspensions from B16 tumor-bearing mice. Representative FACS plots show FoxP3 against IFNγ after pre-gating on $CD3^{+}CD4^{+}$ T cells. Dotplots show frequency of $FoxP3^{+}IFN\gamma^{+}$ cells as mean ± S.E.M. from 6 mice in each group.

Supplementary Figure 6

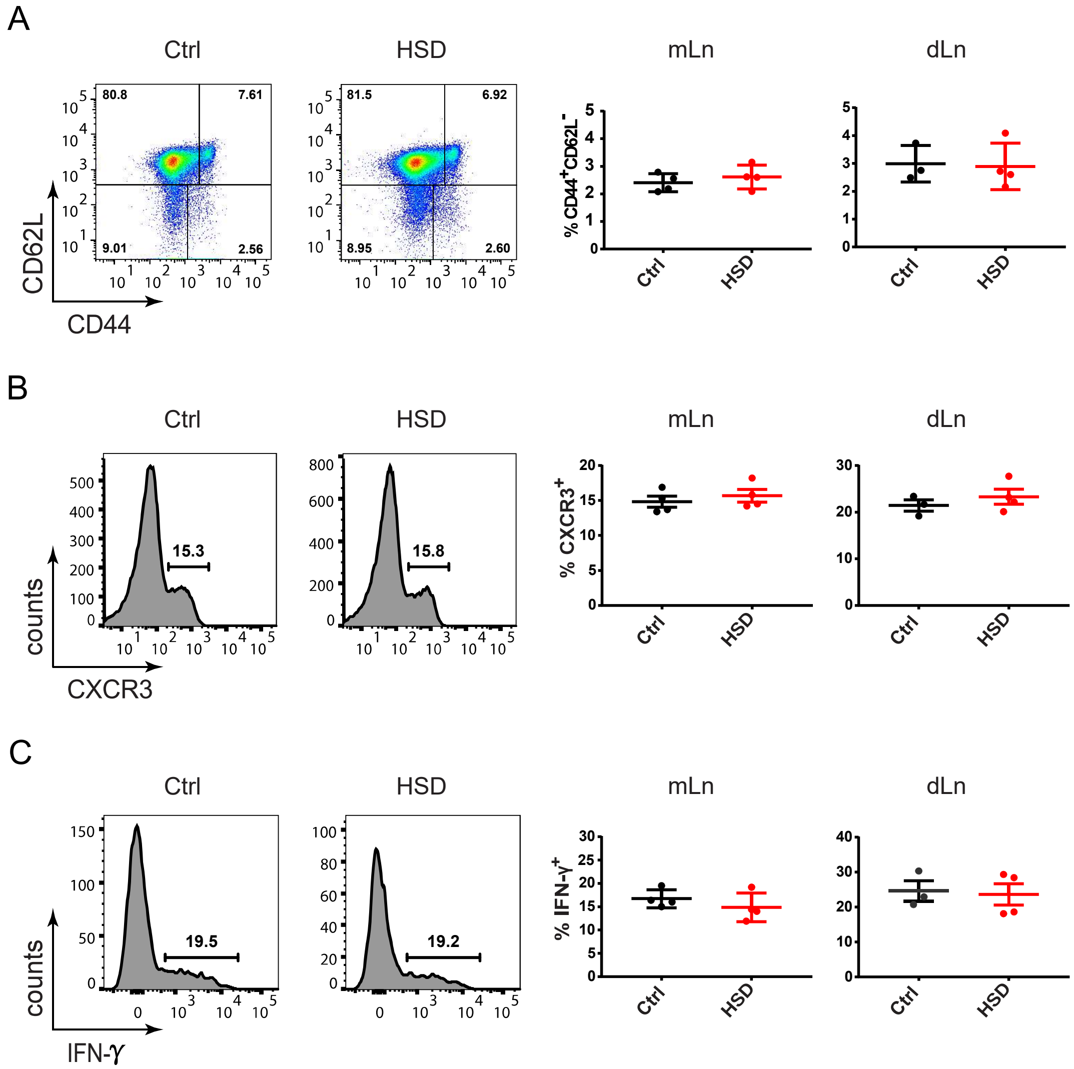

Supplementary Figure 6. Characterization of CD8^{+} T cells in tumor-bearing mice. CD8^{+} T cells were analyzed in mLN and dLN of B16 tumor-bearing control (Ctrl) and high salt fed (HSD) mice. A) FACS plots show CD44 and CD62L expression profile after gating on CD3^{+}CD4^{-} T cells. B) Histogram shows CXCR3 expression on CD3^{+}CD4^{-} T cells. C) Histograms show representative intracellular IFNγ staining of CD3^{+}CD8^{+} T cells after PMA/ionomycin restimulation. Dotplots show frequency as mean ± S.E.M. from 3-4 mice per group. Data is representative for two independent experiments in the B16 and LLC tumor model.

Supplementary Figure 7

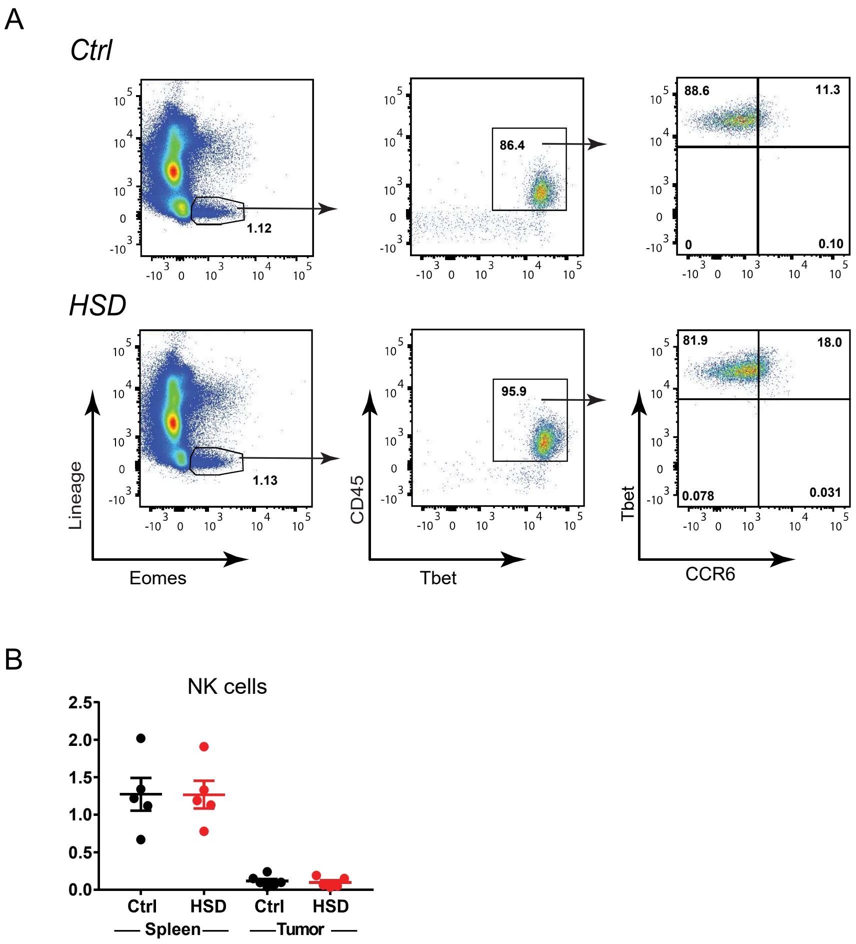

Supplementary Figure 7. Characterisation of NK cells in tumor-bearing mice. Tumors and spleens from B16 tumor-bearing mice were analysed for NK cells by flow cytometry. A) FACS plots show the indicated parameters after pre-gating on singlets and dead cell exclusion. NK cells were defined as lineage (CD3, CD5, CD19, Ly6C/Ly6G)$^-$ and Eomes$^+$T-bet$^+$CD45$^+$CCR6$^-$. Data are representative for spleens of control (upper row) and HSD fed mice (lower row). B) Bar graph shows the frequency of NK cells from tumor and spleen as mean SEM (n=5-6) from one of two independent experiments with similar results.

Supplementary Figure 8

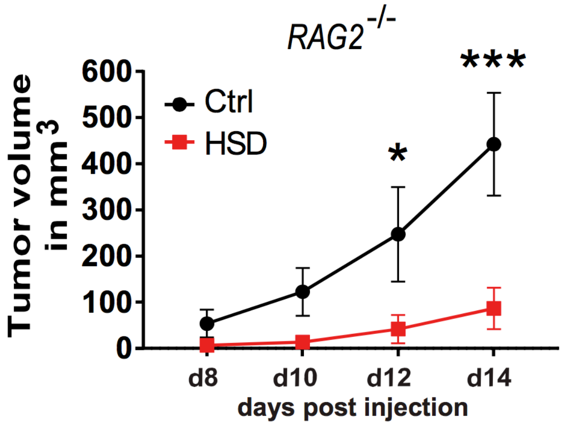

Supplementary Figure 8. High salt inhibits tumor growth in T and B cell deficient mice. As shown for the LLC tumor model (Figure 3g), *RAG2*$^{-/-}$ mice were fed a high salt diet (HSD) or control diet (Ctrl) and challenged with B16 tumor cells. Growth curves show tumor volume as mean ± S.E.M. from 6 mice in each group. Statistical analysis was performed by Two-way repeated measure ANOVA test (*$p<0.05$, ***$p<0.001$).

Supplementary Figure 9

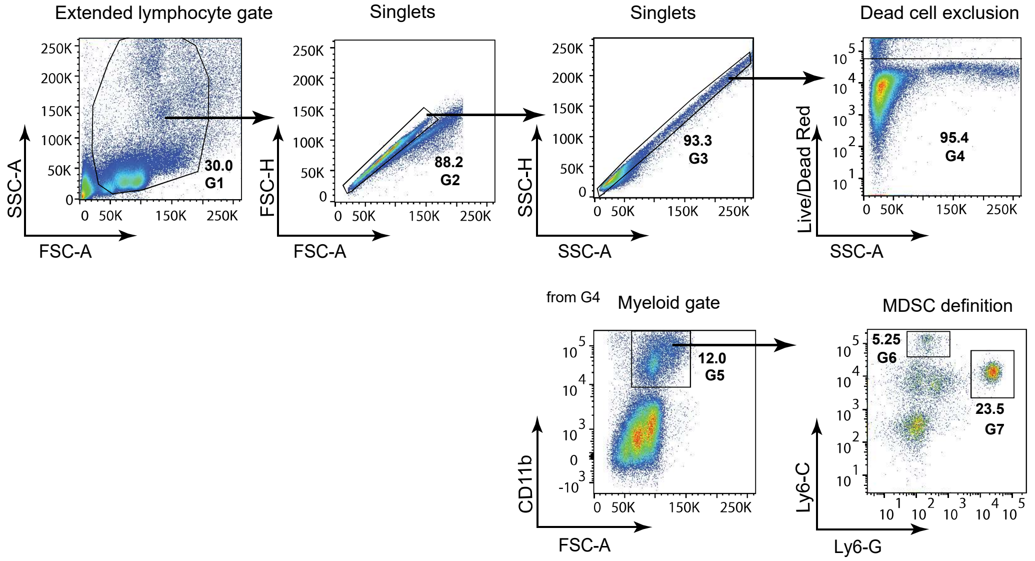

Supplementary Figure 9. Gating strategy for MDSCs in tumor-bearing mice. Representative gating profile for a blood sample from tumor-bearing mice corresponding to Figure 4. Cellular events were defined according to FSC-A and SSC-A profile (G1). Singlets were gated out in two steps including FSC-A/FSC-H (G2) and SSC-H/SSC-A (G3). Dead cells were excluded as positive for the fixable viability dye LD Red (G4). Myeloid cells were defined as CD11b^{+} (G5) and further gated according to Ly6-C and Ly6-G to address MDSC-like populations as M-MDSC: Ly6-C^{high} and Ly6-G^{-} (G6) and PMN-MDSC: Ly6-C^{med} and Ly6-G^{high} (G7). Spleen and tumor-derived single cell suspensions were analyzed in a similar manner including CD45^{+} to pregate on hematopoietic cells.

Supplementary Figure 10

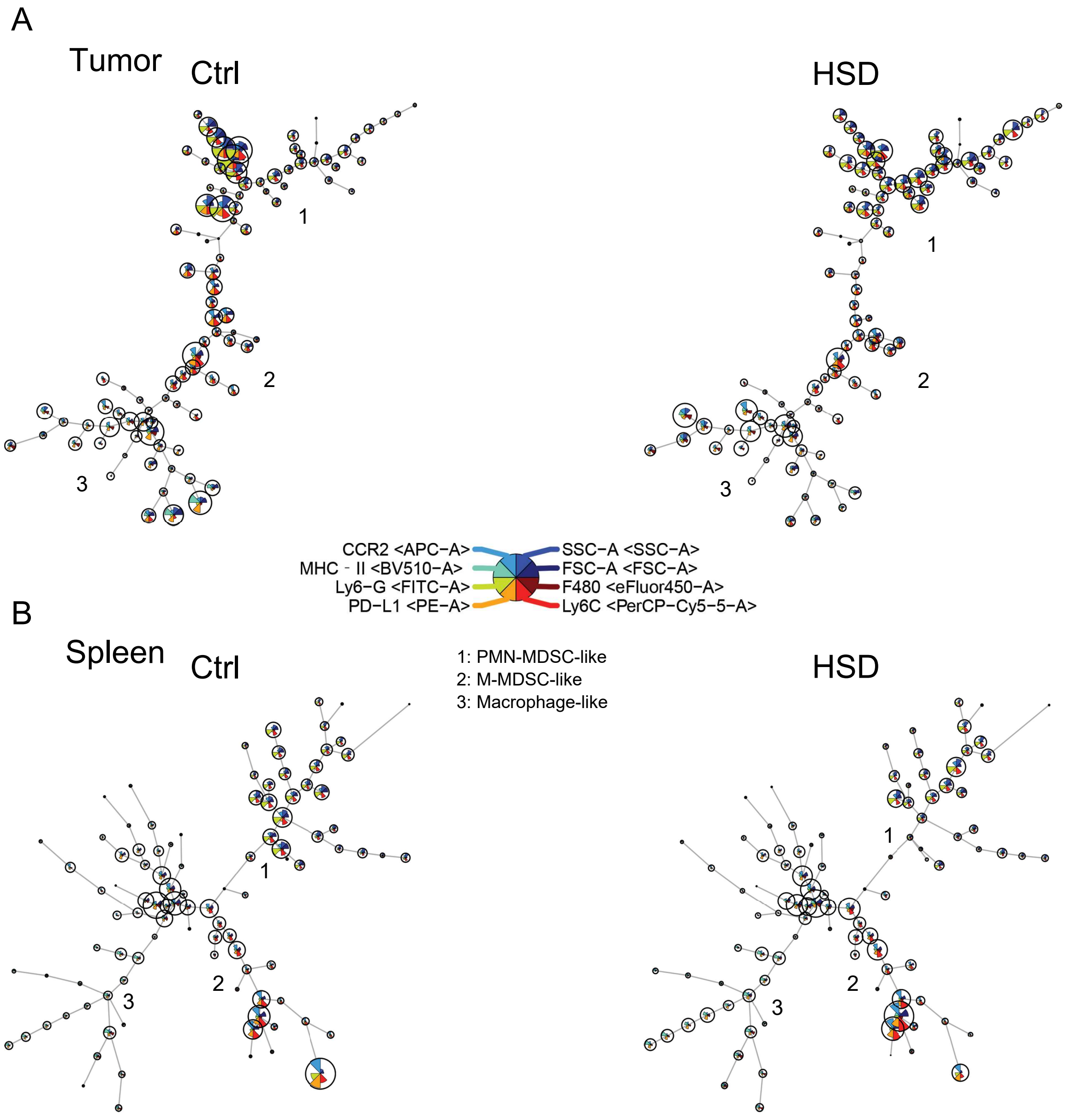

Supplementary Figure 10. Characterization of MDSCs in tumor-bearing mice. A) FlowSOM visualization of flow cytometry data derived from B16 tumor tissue. Single live CD11b^{+} cells for each sample (Ctrl and HSD, n=6/group) were exported and concatenated then analyzed using FlowSOM, which arranges the cells into clusters (represented by circles) according to similarities in their expression profiles. Each node represents one cluster (total = 100 nodes).

B) FlowSOM visualization of flow cytometry data from spleen of B16 tumor-bearing mice. Single live $CD11b^+$ cells for each sample (Ctrl and HSD, n=6/group) were exported and concatenated then analyzed using FlowSOM, which arranges the cells into clusters (represented by circles) according to similarities in their expression profiles. Each node represents one cluster (total = 100 nodes).

Supplementary Figure 11

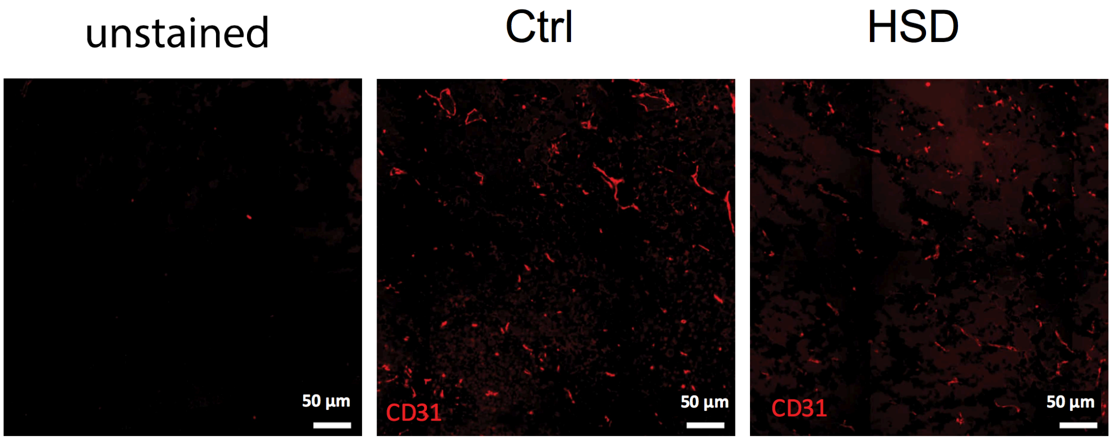

Supplementary Figure 11. Immunohistochemistry for CD31 in tumor tissues. Unstained controls (left) and anti-CD31 mAb stainings of LLC tumor tissues from control (center) and HSD (right) fed mice (scale bars = 50µm). Stainings were performed from 3 tumors per group with 3 to 4 areas analyzed. Shown are representative examples.

Supplementary Figure 12

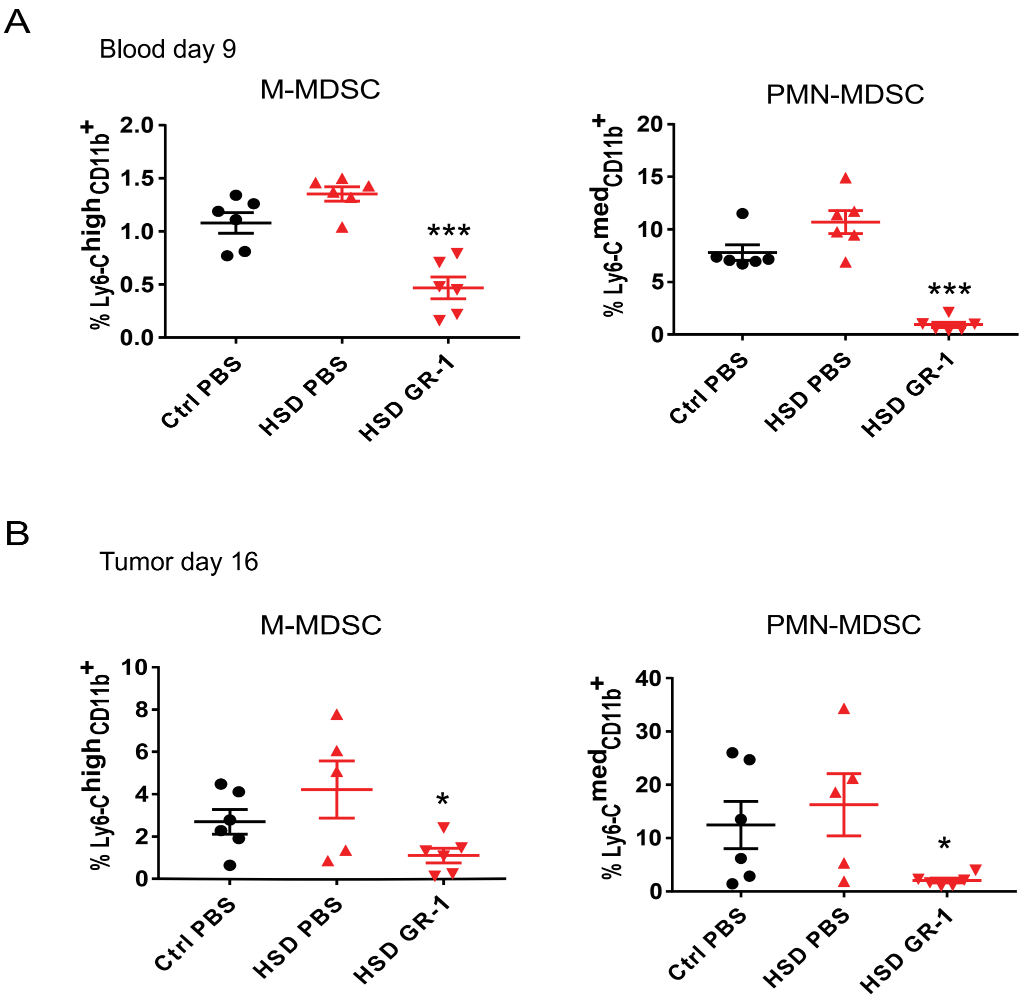

Supplementary Figure 12. Depletion of MDSCs by anti-Gr1 antibodies in tumor-bearing mice. High salt diet (HSD) fed mice were treated with anti-Gr-1 antibody or PBS as control from day 4 after B16 melanoma cell inoculation on consecutively every second day. Mice were monitored for depletion efficiency in blood 5 days after receiving the first antibody injection (day 9) and finally in tumors at day 16 p.i.. Bar graphs show the frequencies of $CD11b^{+}Ly6\text{-}C^{high}$ M-MDSCs and $CD11b^{+}Ly6C^{med}$ PMN-MDSCs as mean ± S.E.M. from 5-6 mice in each group. Statistical significance between the groups HSD PBS & HSD GR-1 was determined by t-test (*$p<0.05$, ***$p<0.001$).

Supplementary Figure 13

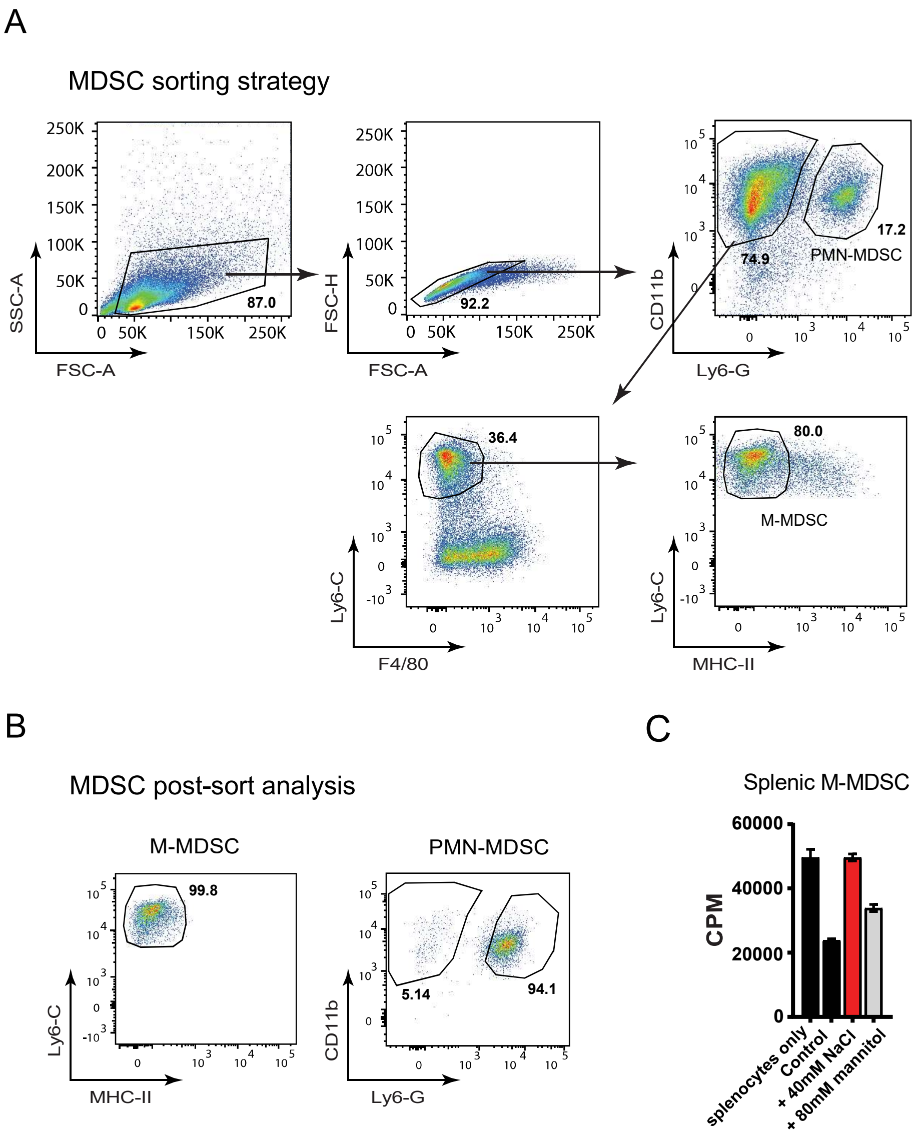

Supplementary Figure 13. Sorting of MDSCs from tumor-bearing mice and *in vitro* suppression assay. MDSCs were sorted from tumors and spleens from LLC tumor-bearing mice for T cell suppression assays as in Figure 5. A) Debris and dead cells were excluded by SSC-A and FSC-A gating. Singlet cells were discriminated by FSC-A versus FSC-H profile. PMN-MDSCs were defined as $CD11b^{+}Ly6\text{-}G^{+}$, M-MDSCs were defined as $CD11b^{+}Ly6\text{-}C^{+}Ly6\text{-}G^{-}F4/80^{-}MHCII^{-}$.

B) Representative FACS plots from post-sort analysis for M-MDSCs and PMN-MDSCs are shown.

C) Sorted splenic M-MDSC were analyzed for their capacity to suppress T cell proliferation in the presence of additional 40mM NaCl or 80mM Mannitol as osmolyte control. Bar graphs show proliferation (CPM) as measured by 3H-thymidine incorporation of responder cells alone or in the presence of MDSCs at a MDSC/T cell ratio of 1:1 under indicated conditions as mean ± S.E.M. (n=3). Data is representative of two independent experiments.

Supplementary Figure 14

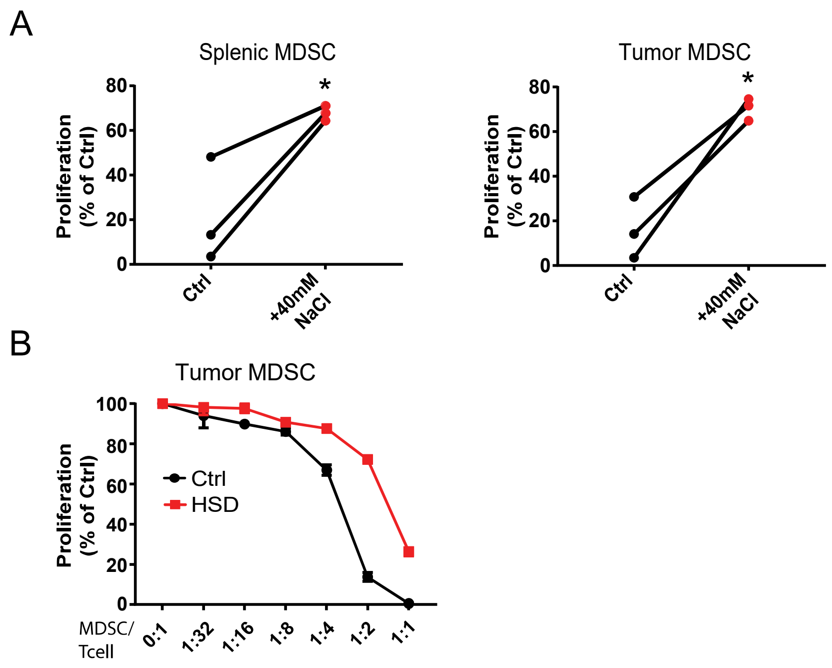

Supplementary Figure 14. High salt blocks suppressive function of MDSCs. A) Suppression assay from three independent experiments performed with splenic MDSC cultured in ratio of 1:1 (left) or tumor-derived MDSCs at a ratio of 1:2 (right) with responder splenocytes as shown in Figure 5a. Statistical significance was determined by t-test (*$p<0.05$). B) MDSCs were FACS sorted from LLC tumors either derived from high salt diet (HSD) or control (Ctrl) diet fed mice and co-cultured with splenocytes at the indicated ratio without adding additional NaCl *in vitro*. Proliferation of responder splenocytes was measured by 3H-thymidin incorporation. Curves show proliferation was normalized to controls (stimulated splenocytes without addition of MDSCs) and data is shown as mean ± S.E.M from triplicates.

Supplementary Table 1

sex	age	Localisation	T-stage	N-stage	M-stage
male	62	Oropharnyx	2	0	0
male	59	Oropharnyx	3	2	x
female	53	Oropharnyx	1	0	0
male	58	Oropharnyx	2	1	1
male	68	Bladder	a (not invasive)		

Supplementary Table 1. Patient characteristics. Characteristics of cancer patients with bladder cancer (1) and head and neck cancer (4) that were tested for the effects of high salt conditions on MDSC function *in vitro*.
